# Genomics Analyses Reveal Unique Classification, Population Structure and Novel Allele of Neo-Tetraploid Rice

**DOI:** 10.1186/s12284-021-00459-y

**Published:** 2021-02-06

**Authors:** Hang Yu, Qihang Li, Yudi Li, Huijing Yang, Zijun Lu, Jinwen Wu, Zemin Zhang, Muhammad Qasim Shahid, Xiangdong Liu

**Affiliations:** 1 State Key Laboratory for Conservation and Utilization of Subtropical Agro-Bioresources, South China Agricultural University, Guangzhou, 510642 China; 2 Guangdong Provincial Key Laboratory of Plant Molecular Breeding, South China Agricultural University, Guangzhou, 510642 China; 3 College of Agriculture, South China Agricultural University, Guangzhou, 510642 China; 4 Guangdong Laboratory for Lingnan Modern Agriculture, South China Agricultural University, Guangzhou, 510642 China

**Keywords:** *Oryza sativa*, Neo-tetraploid rice, Genetic diversity, Population structure, Novel DNA variation

## Abstract

**Background:**

Neo-tetraploid rice (NTR) is a useful new germplasm that developed from the descendants of the autotetraploid rice (ATR) hybrids. NTR showed improved fertility and yield potential, and produced high yield heterosis when crossed with *indica* ATR for commercial utilization. However, their classification, population structure and genomic feature remain elusive.

**Results:**

Here, high-depth genome resequencing data of 15 NTRs and 18 ATRs, together with 38 publicly available data of diploid rice accessions, were analyzed to conduct classification, population structure and haplotype analyses. Five subpopulations were detected and NTRs were clustered into one independent group that was adjacent to *japonica* subspecies, which maybe the reason for high heterosis when NTRs crossed with *indica* ATRs. Haplotype patterns of 717 key genes that associated with yield and other agronomic traits were revealed in these NTRs. Moreover, a novel specific SNP variation was detected in the first exon of *HSP101,* a known heat-inducible gene, which was conserved in all NTRs but absent in ATRs, 3KRG and RiceVarMap2 databases. The novel allele was named as *HSP101–1*, which was confirmed to be a heat response factor by qRT-PCR, and knockout of *HSP101–1* significantly decreased the thermotolerance capacity of NTR. Interestingly, *HSP101–1* was also specifically expressed in the anthers of NTR at pre-meiotic and meiosis stages under optimal environment without heat stress, and its loss-of-function mutant showed significant decrease in fertility of NTR.

**Conclusion:**

The construction of first genomic variation repository and the revelation of population structure provide invaluable information for optimizing the designs of tetraploid rice breeding. The detection of specific genomic variations offered useful genomic markers and new directions to resolve high fertility mechanism of NTR.

**Supplementary Information:**

The online version contains supplementary material available at 10.1186/s12284-021-00459-y.

## Background

Whole-genome duplication (WGD) or polyploidization played vital role in evolution, speciation and biological complexity of higher plants (Van de Peer et al. 2009). Polyploids, including autotetraploids and allopolyploids, are widespread in flowering plants and 60%–70% flowering plants have one or more polyploid ancestor (Blanc and Wolfe 2004; Masterston 1994). The advantage of polyploids in plant growth, yield and environmental fitness prompt the crop breeders to explore their usage in modern crop trait improvement (Van de Peer et al. 2009; Renny-Byfield and Wendel 2014).

Autotetraploid rice is a germplasm that generated by artificial genome duplication of diploid rice and their development enriched the germplasm resources of rice (Tu et al. 2003; Luan et al. 2007; Cai et al. 2007; Lu et al. 2020). Although biological advantages, wide adaptability and high hybrid vigor were observed in autotetraploid rice (Shahid et al. 2011; Shahid et al. 2012; Wu et al. 2013; Tu et al. 2014; Yang et al. 2014), their production and commercial utilization were restricted by many factors, especially fertility (Shahid et al. 2010; He et al. 2011; Shahid et al. 2013; Wu et al. 2014; Wu et al. 2015; Li et al. 2017; Chen et al. 2018; Li et al. 2018). After many years of tremendous efforts, a newly developed tetraploid rice with high fertility, i.e. neo-tetraploid rice, was developed by our research group through crossing of autotetraploid rice (Guo and Liu 2014; Guo et al. 2016; Guo et al. 2017; Bei et al. 2019; Yu et al. 2020; Ghaleb et al. 2020), which may become new useful resources for rice breeding (Koide et al. 2020). Compared with ATR, several agronomic traits improved significantly in NTR, such as seed setting and grain number per panicle, and also contained non-parental variant genes associated with fertility and yield (Yu et al. 2020). Hybrids between NTR and ATR showed high heterosis and production (Guo et al. 2017; Ghaleb et al. 2020). Consequently, genetic diversity between ATR and NTR populations need to be assessed urgently to illustrate the population structure and genetic divergence.

The revelation of genetic divergence and population structure is very important either in maximizing hybrid vigor, optimizing breeding design or discovering trait associated variations. The population structure in plant species, such as wheat (Cavanagh et al. 2013; Chao et al. 2010; Maccaferri et al. 2005), maize (Romay et al. 2013), sorghum (Morris et al. 2013), barley (Malysheva-Otto et al. 2006), pearl millet (Serba et al. 2019) and weedy rice (De Leon et al. 2019), were illustrated using microsatellites or simple sequence repeat (SSR) markers and single-nucleotide polymorphisms (SNPs). With the development of next-generation sequencing, whole genome re-sequencing was used as a valid method to conduct genotyping in populations to analyze the genetic diversity. In diploid rice, population structure analysis based on genome re-sequencing data classified 3010 diverse accessions into nine subpopulations, which was accordant with their geographic distribution (Wang et al. 2018b).

Based on the genotypic data of population, haplotype analysis can be used to illustrate the genetic diversity. Haplotype analysis of genes that regulate important agronomic traits has been used to identify trait associated variations and functional genetic markers. The SNP variations in *BADH1* gene were detected by high-throughput SNP genotyping method from 16 varieties differing in salt tolerance and aroma (Singh et al. 2010). Haplotype analysis of grain weight gene, *GW2*, in diverse *indica* and aromatic genotypes discovered four new SNPs and regrouped the haplotypes into four categories (Dixit et al. 2013). In another study, haplotype analysis of 21 salt stress-responsive candidate genes in 103 wild rice accessions with contrasting salt tolerance was conducted, which revealed the major alleles in different geographic regions (Mishra et al. 2016). Haplotype analysis using deep genome re-sequencing is becoming a reliable method to inspect genetic diversity. Genome re-sequencing of 104 rice varieties provided 18 million variations for genetic diversity analysis and haplotype reconstruction of agronomically important genes (Duitama et al. 2015). Using the sequencing data of 3 K rice genome panel, haplotypes of 120 genes regulating grain yield and grain quality were explored, and the assessment of superior haplotypes showed potential in developing next-generation tailor-made rice (Abbai et al. 2019). The construction of haplotype patterns of agronomically important genes in tetraploid rice, based on the previously reported genes in diploid rice, would provide valuable resources for their functional analysis.

In the present study, the genomic re-sequencing data of neo-tetraploid rice and autotetraploid rice lines was used to reveal the genome features, population structure and haplotype patterns. By these analyses, we planned (1) to determine the subspecies (*indica*-*japonica*) classification and population structure of neo-tetraploid rice, (2) to construct the haplotype patterns of agronomically important genes in tetraploid rice population, (3) to mine the novel specific alleles of neo-tetraploid rice, and (4) to validate the function of neo-tetraploid rice specific genes.

## Results

### Genome Re-Sequencing and Variation Detection in Neo-Tetraploid and Autotetraploid Rice

The genome re-sequencing data of 15 neo-tetraploid rice lines (NTRs) and 18 autotetraploid rice lines (ATRs) with about 46-fold sequencing depth were used in this study, which included about 2 billion high quality pair-end sequencing reads with an average ratio of Q30 score of 93.98% (Table S1). The average genome coverage ratios of MSU7 and R498 reference genome were 95.31% and 93.46%, respectively (Table S2). About 66.9 million variations, ranged from 0.21 to 3.50 million variations in each individual, were identified in 33 tetraploid rice (including ATR and NTR) lines when compared with MSU7 reference genome. Approximately 85.7 million variations, ranged from 1.26 to 3.44 million variations in each individual, were identified in 33 tetraploid rice (including ATR and NTR) lines when mapped onto R498 reference genome (Table S3).

The genomic variations from 33 tetraploid rice lines were combined based on their genomic positions, and a total of 7,445,008 and 7,880,885 variations were detected between 33 tetraploid rice lines and reference genomes of MSU7 and R498, respectively. A total of 0.79 million moderate-to-high effect variations were identified based on the annotation results from SnpEff software, which accounted for 10.61% of the total variations against MSU7 reference genome. The percentage of modifier and low variations were 6.96% and 82.42%, respectively (Table S4).

Variation density analysis revealed 829.28, 385.75, 304.89 and 501.01 variations (including SNPs and InDels) on an average per 100 Kb in *indica* ATRs, *javanica* ATRs, *japonica* ATRs and NTRs against MSU7 reference genome, respectively. Against R498 reference genome, with an average of 477.70, 797.53, 766.37 and 713.31 variations (including SNPs and InDels) per 100 Kb in *indica* ATRs, *javanica* ATRs, *japonica* ATRs and NTRs were detected, respectively (Table S5). The distributions of variation density were illustrated using “Circos” plot (Fig. S1 and Fig. S2), and low-diversity regions (LDRs) were found in Chr5 (10–14 Mb) and Chr6 (2–6 Mb). These LDRs contained relatively low number of variations in *indica* ATRs, *japonica* ATRs, *javanica* ATRs and neo-tetraploid rice (NTRs) against two reference genomes.

### Genomic Classification and Population Structure Analysis of Neo-Tetraploid Rice

**Fig. 1:**
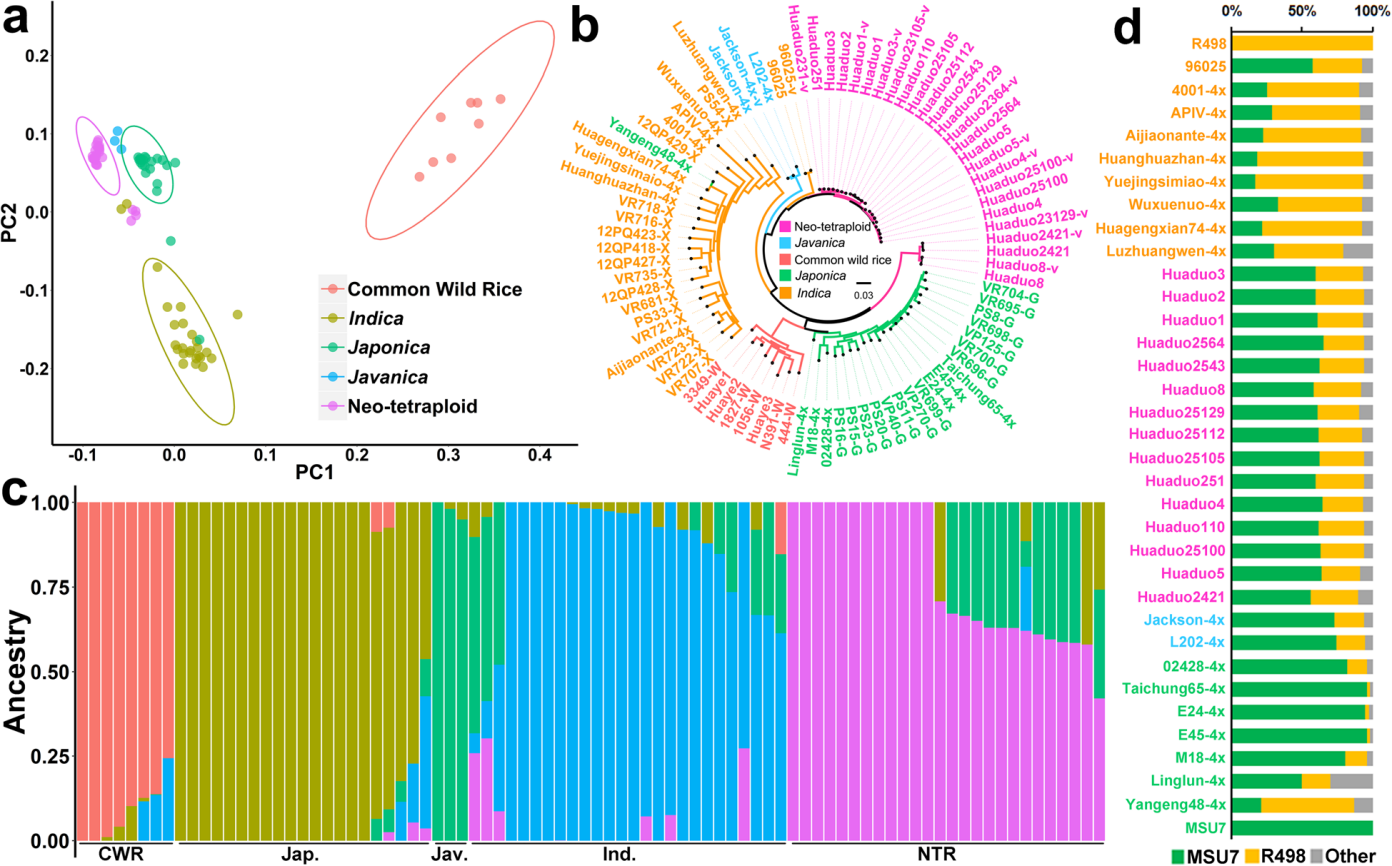
Population structure analysis of tetraploid rice lines. PCA (**a**) and phylogenetic (**b**) analyses of tetraploid rice population based on whole genome SNP variations, (**c**) Population structure analysis conducted by ADMIXTURE with *k* = 5, (**d**) Fraction of *indica* (R498) and *japonica* (MSU7) genomes present in autotetraploid rice (ATRs) and neo-tetraploid rice (NTRs). Sample names start with “Huaduo” are NTRs. Accession 96,025 and the accessions with the extensions of “-4x” are ATRs, and samples from different ecotypes were distinguished by color. Samples with extensions of “-G”, “-I” represents *japonica* and *indica* diploid rice, respectively. Common wild rice samples start with “Huaye” or end with “-W”

There are large genetic divergences between the genomes of the two Asian cultivated rice (*Oryza sativa* L.) subspecies, *indica* and *japonica*. Mostly, the mapping rate is high and variations are less when the sequencing sample and the reference genome belonging to the same subspecies. Subspecies classification of NTRs was detected using the difference of mapping rate and genomic variation number between NTRs and two reference genomes (*japonica* reference genome, MSU7, and *indica* reference genome, R498). With minimal coverage of 5X, the average mapping rates were 92.39% and 89.13% with MSU7 and R498 in all NTRs, respectively. On the contrary, the average variation numbers of all NTRs were 1,903,495 and 2,818,017 against MSU7 and R498, respectively. The tendency was consistent with the known *japonica* materials in the previous studies, which suggested that the genome sequences of NTRs were similar to *japonica* (Fig. S3 and Fig. S4). Moreover, to identify the fractions of *indica* and *japonica* genomes present in ATRs and NTRs, 2,785,296 SNPs and InDels between MSU7 and R498 were used as markers. The results were consistent with the genome coverage and variation number, which also showed that NTRs have more fraction of MSU7 genomes than R498 (Fig. 1d). The classification was further validated using phenol reaction according to Cheng’s index, and the glume color of *indica* ATR, Huanghuazhan-4x, and its diploid counterpart were dyed to dark purple, while NTRs, *japonica* ATR Taichung65-4x and its diploid counterpart were unstained (Fig. S5). These results indicated that NTRs can be classified into *japonica* subspecies.

Together with 38 published genome sequencing accessions, principal component analysis (PCA) and phylogenetic relationship analysis classified all materials into five different groups, namely *Oryza rufipogon* (common wild rice), *indica*, *japonica*, *javanica* (tropical *japonica*) and NTR, and the classifications of most samples were in accordance with their known subspecies features (Table S1, Fig. 1a, b). To validate the population structure results, the ADMIXTURE analysis was conducted in the *k* values (subgroup number) ranging from 2 to 8, and *k* = 5 was chosen as the best subgroup number because the lowest cross-validation error was detected when *k* = 5 (Fig. 1c, Table S6). These results indicated that the NTRs can be classified into an independent subgroup in tetraploid rice population. The independent position of NTRs compared to other type of tetraploid lines, especially *indica* autotetraploid lines, demonstrated the potential high heterosis effect between NTRs and ATRs, which was consistent with our previous results of F_1_ hybrid’s performance (Yu et al. 2020). Notably, genomic data also revealed that all NTRs contained wide compatibility gene *S*_*5*_^*n*^, which would play important roles in the breeding of intersubspecific tetraploid rice hybrids (Fig. S6).

### Haplotype Analysis in Tetraploid Rice Population

Haplotype pattern of 55,801 annotated genes in rice whole genome (MSU7) was determined to evaluate the genetic diversity of tetraploid rice population. A total of 432,290 haplotypes were identified, with an average of 7.75 haplotypes per gene. The distribution of number of haplotypes per gene revealed that 40,013 out of 55,801 genes have 2 to 15 haplotypes (Fig. S7). However, 7772 genes only have one haplotype. GO enrichment analysis revealed that those zero-diversity genes participated in the basic key metabolic processes like GTP binding, proton-transporting ATPase activity and NADH dehydrogenase activity (Table S7).

In order to harness the genetic diversity of tetraploid rice for breeding, the haplotype variations of previously cloned genes associated with yield and other agronomically important traits were identified in tetraploid rice population (Table S8). In 717 agronomically important genes, 2912 haplotypes were detected, with an average of 4.06 haplotypes per gene (Table S9). The distribution of haplotypes per gene revealed that 563 out of 717 genes have 2 to 15 haplotypes per gene, while 147 genes have only one haplotype in tetraploid rice accessions (Fig. S8). For instance, the yield related genes *Hd3a* (*LOC_Os06g06320*), *NAL1* (*LOC_Os04g52479*) and *sd1* (*LOC_Os01g66100*) have 4, 6 and 9 haplotypes, while *TAC1* (*LOC_Os09g35980*) gene only have one haplotype.

### Identification of Novel Specific Alleles in Neo-Tetraploid Rice

**Fig. 2:**
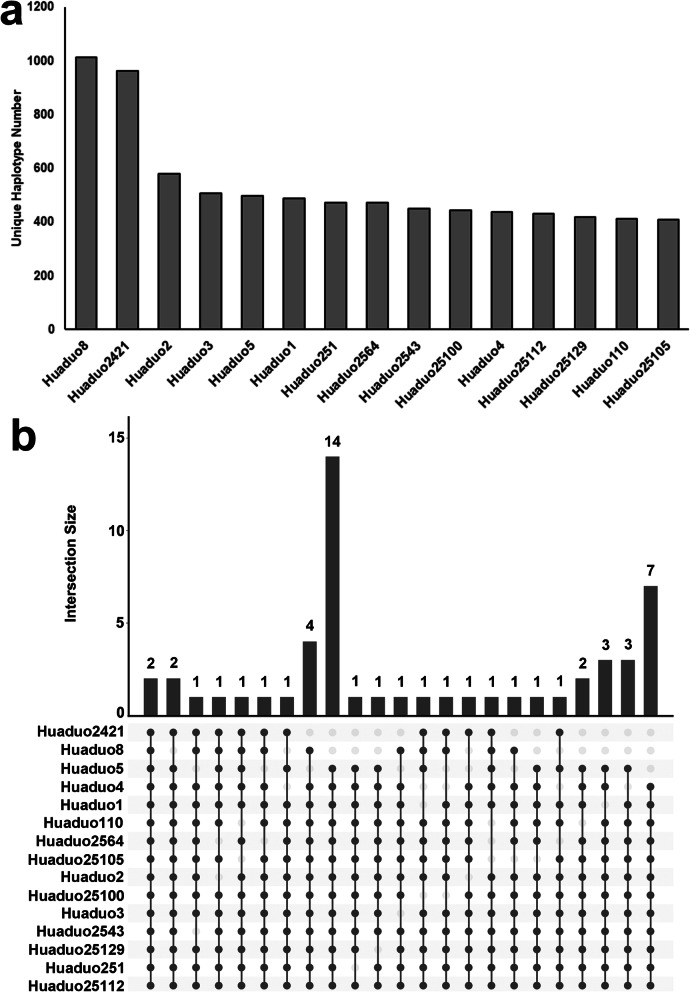
Number of genes that contained specific haplotypes in 15 neo-tetraploid rice (NTR) lines. **a** Number of specific genes in 15 neo-tetraploid rice lines when compared with autotetraploid rice lines, **b** UpSet plot shows the intersections with intersection size larger than 12. The dot in the bottom indicates the intersections. The bar plot in the top showed the haplotype number in each intersection set

The results of haplotype analysis were further used to identify NTR specific alleles compared to ATRs. The haplotype patterns of 55,801 annotated genes of MSU7 reference genome among 18 ATRs, 15 NTRs and 30 diploid rice lines (DRs) were compared. A total of 269 shared unique haplotypes were found in the NTRs, but no unique haplotype was detected in the ATRs when compared with the DR genomes, suggesting that NTRs may contain novel variations, which is neither present in DRs, nor in ATRs (Table S10). Moreover, the independent position of population structure and significant genetic differences between NTR and ATR populations prompted us to analyze their allele differentiation. Firstly, the haplotypes of 15 NTRs were compared with 18 ATRs one by one, and 3189 specific haplotypes were identified in 15 NTRs. Huaduo 8 and Huaduo 2421 contained 1014 and 964 specific haplotypes, respectively, which were higher than other NTRs (Fig. 2a). The intersections of haplotypes in each NTR line were analyzed by UpSet plot, and the numbers of haplotypes that shared by more than 12 NTRs are shown in Fig. 2b. The two haplotypes, which shared by all 15 NTR lines, and 14 haplotypes that shared by 13 NTR lines, were identified.

**Fig. 3:**
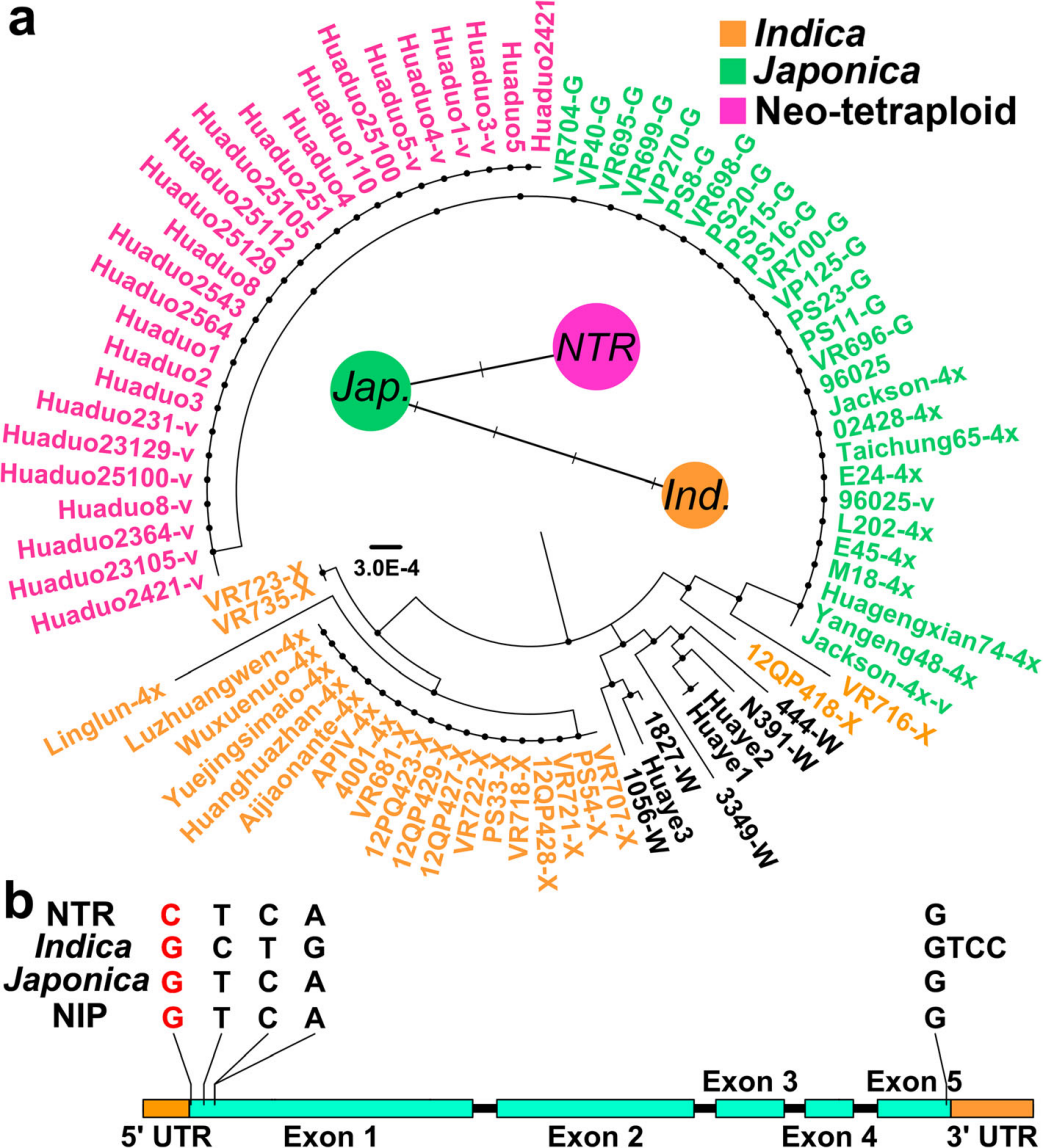
The phylogenetic tree and haplotype network of *HSP101* in tetraploid rice population (**a**), and illustration of gene structure and neo-tetraploid specific novel variation (**b**). NTR specific base substitutions were highlighted with red font

One allele was considered as NTR specific allele when all the haplotypes of NTRs were uniform but different from ATRs. Five NTR specific haplotypes were identified, and two of them have homozygous variations, including *LOC_Os05g44340* (*HSP101*, heat response gene) and *LOC_Os10g25730* (hypothetical protein) (Fig. 3a, Fig. S9). Of these five genes, one gene, *HSP101*, was highly expressed in anther at meiosis stage in NTR Huaduo 1 under natural environment (Fig. S10a). One base substitution was detected in *HSP101* of all NTR lines but not in ATRs. The allele distribution of *HSP101* in 18 ATRs and 35 DRs were further investigated, after removing the low-quality sequenced materials, the allele of *HSP101* could be divided into two types, namely *indica* (*HSP101-i*) and *japonica* (*HSP101-j*) types. However, *HSP101* allele was different and uniform in all NTRs, with a unique base substitution from G to C that caused a missense mutation in exon 1 and amino acid change from proline to alanine (Fig. 3b). Haplotype network analysis revealed that novel allele of *HSP101* has a closer relationship to the *japonica* haplotype of *HSP101* (Fig. 3a). This variation was validated by sanger sequencing in 15 NTRs and 15 ATRs (Table S11), while the variation was not detected in diploid rice lines of the RFGB (Table S12) and RiceVarMap2 (Table S13) databases, which suggested that it is a novel specific variation in NTRs. Therefore, this haplotype was named as “*HSP101–1*”.

### Function Analysis of *HSP101–1* in Seedling Thermotolerance and Fertility of Neo-Tetraploid Rice

**Fig. 4:**
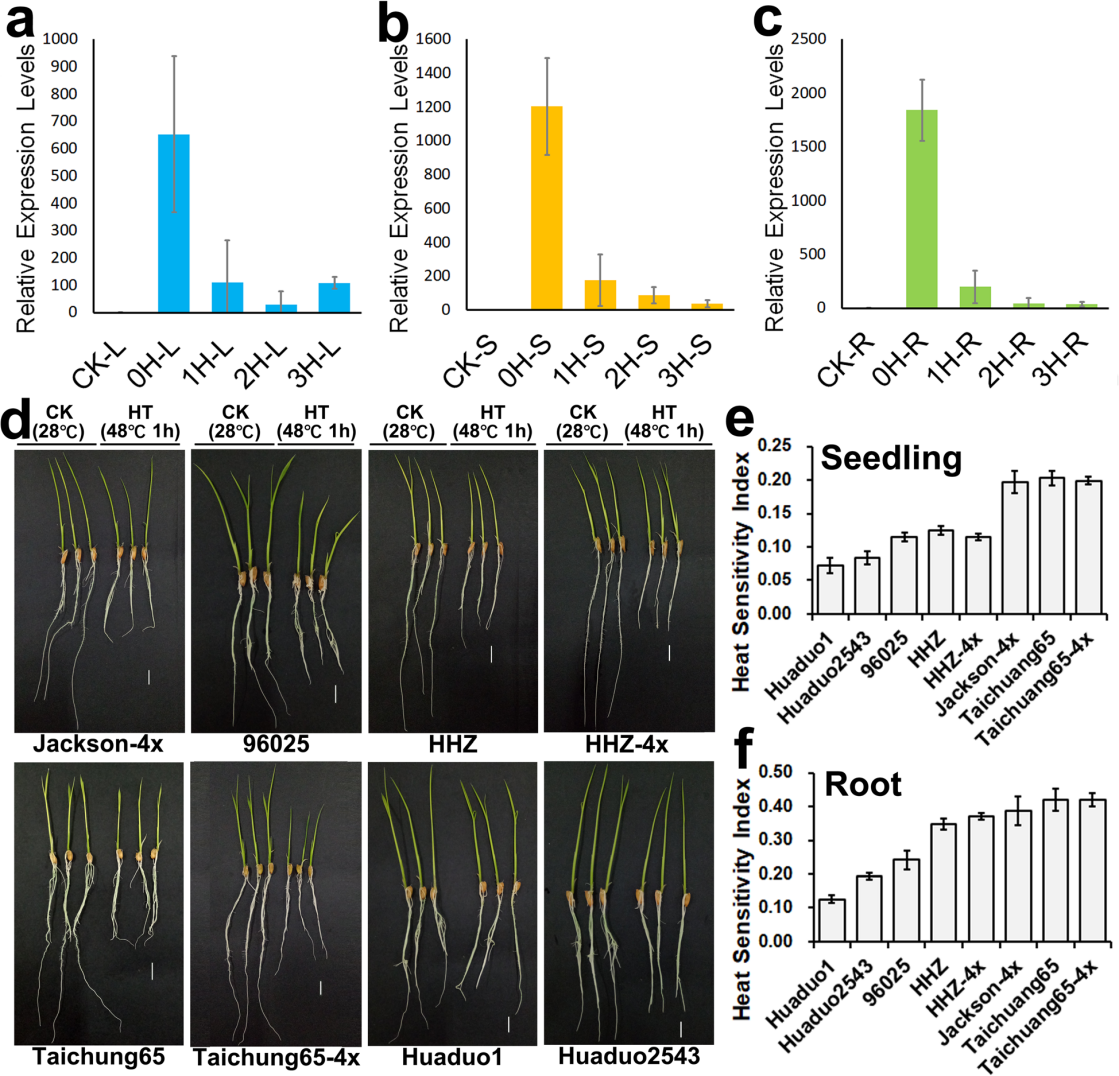
Thermotolerance evaluation of NTRs and ATRs at seedling stage and the expression levels of *HSP101* under heat stress in NTR. **a**, **b**, **c** The expression levels of *HSP101* in response to heat stress in leaf, leaf sheath and root of NTR, Huaduo 1, at seedling stage. **d** The growth of seedlings under high temperature in neo-tetraploid rice lines (Huaduo 1 and Huaduo 2543), *indica* (Huanghuazhan and Huanghuazhan-4x), *japonica* (Taichuang65 and Taichuang65-4x) and NTR parental lines as controls. The scale bar indicates 1 cm. (e, f) Heat sensitivity index of seedling length and root length of all tested lines

**Fig. 5:**
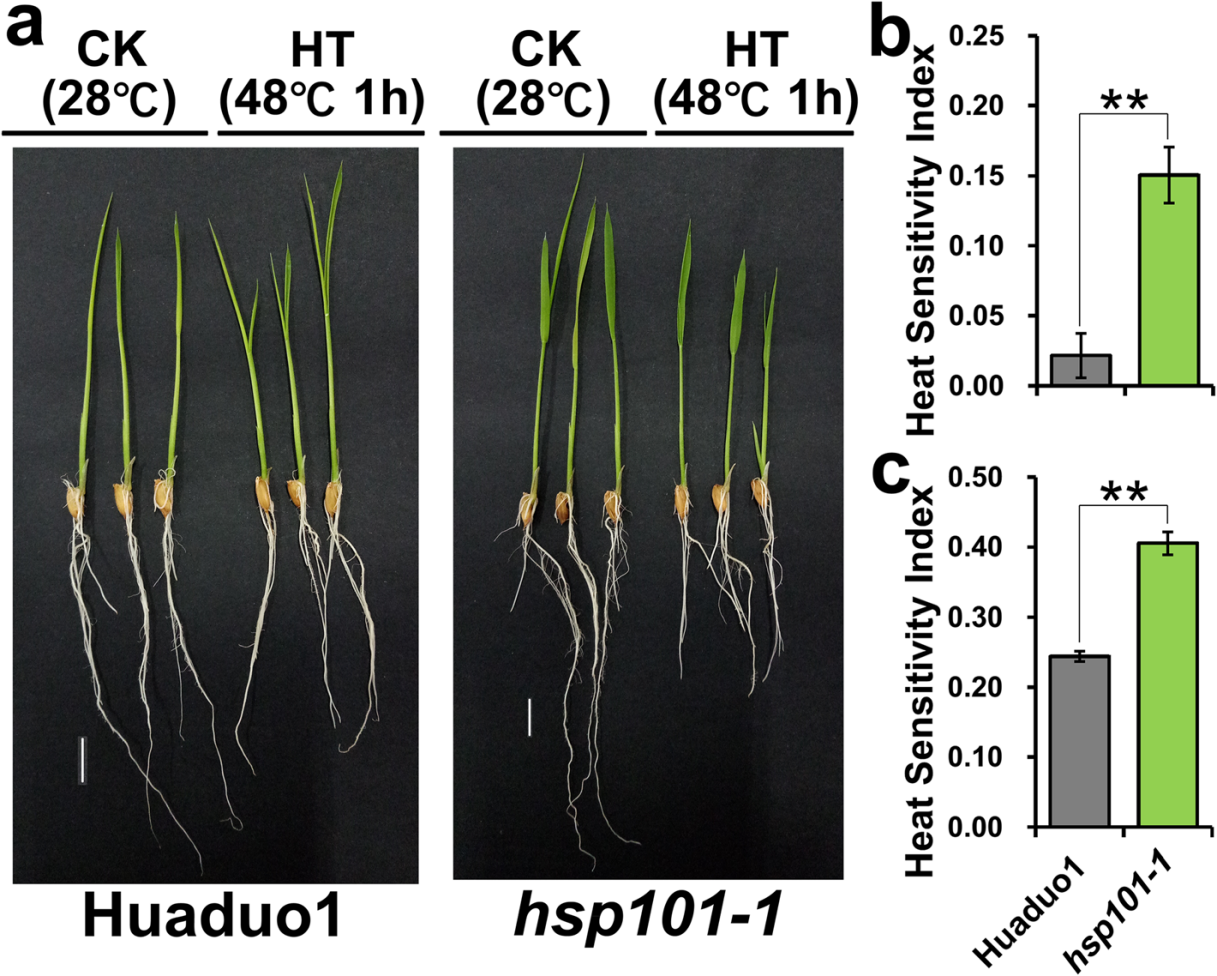
Thermotolerance evaluation of *HSP101–1* knockout mutant. **a** Thermotolerance ability of wild type Huaduo 1 and *hsp101–1* at seedling stage. **b**, **c** Heat sensitivity index of seedling length and root length of Huaduo 1 and *hsp101–1*

The function of *HSP101–1* in neo-tetraploid rice was first validated at seedling stage. qRT-PCR results displayed that *HSP101–1* was up-regulated in leaf, sheath and root at seedling stage in Huaduo 1 after heat treatment, and the expression was up-regulated by 653.19-, 1204.37- and 1843.17- fold change in leaf, sheath and root, respectively. While, the expression decreased gradually after heat shock in Huaduo 1 (Fig. 4a, b and c). These results suggested that *HSP101–1* is a heat stress response factor in neo-tetraploid rice. To evaluate the heat-tolerance of materials with haplotypes of *HSP101–1*, thermotolerance experiment was conducted at seedling stage using 2 NTR lines (*HSP101–1*), Huaduo 1 and Huaduo 2543, and Huanghuazhan (*HSP101-i*), Huanghuazhan-4x (*HSP101-i*), Taichung 65 (*HSP101-j*), Taichung 65-4x (*HSP101-j*), and the NTR parental lines, Jackson-4x (*HSP101-j*) and 96,025 (*HSP101-j*), were used as controls. The results showed that NTRs with the haplotype of *HSP101–1* have enhanced thermotolerance capability and produced stronger seedlings than all controls. The heat sensitivity indexes of seedlings were 0.072 and 0.084 in Huaduo 1 and Huaduo 2543, while the heat sensitivity index in controls were ranged from 0.115 to 0.203, with an average value of 0.159. The heat sensitivity indexes of roots were 0.126 and 0.193 in Huaduo 1 and Huaduo 2543, while the heat sensitivity index in controls were ranged from 0.242 to 0.419, with an average value of 0.365 (Fig. 4d, e and f). To further validate the function, *HSP101–1* was knocked out in neo-tetraploid rice Huaduo 1. The thermotolerance capability of knockout line decreased significantly, and heat sensitivity indexes decreased by about 0.13 and 0.16 in seedling and roots of *hsp101–1*, respectively (Fig. 5).

**Fig. 6:**
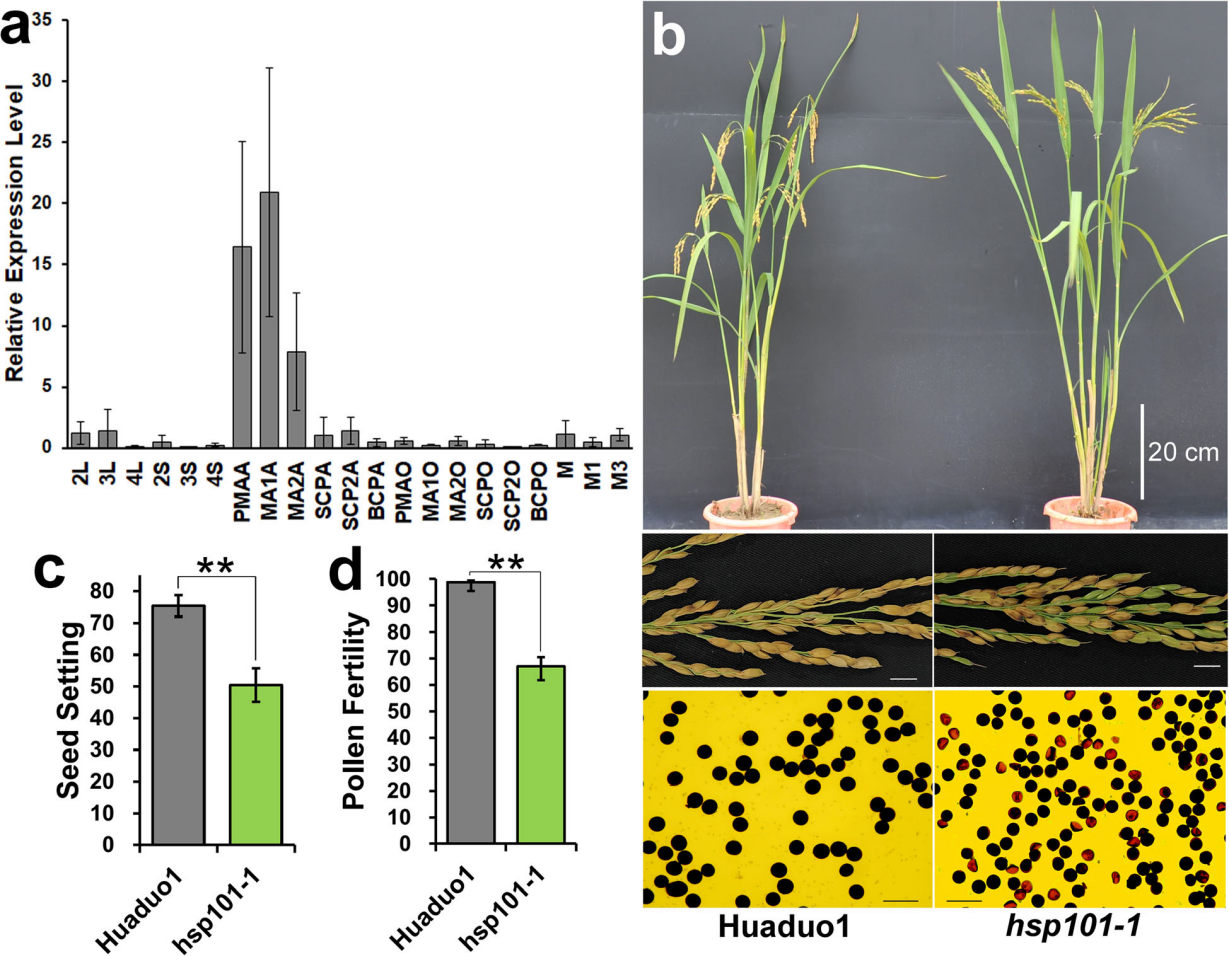
Impact of *HSP101–1* on the fertility of neo-tetraploid rice. **a** Expression pattern analysis of *HSP101–1* in neo-tetraploid rice Huaduo 1 using qRT-PCR. **b** Plant morphology, panicle and pollen fertility of wild type Huaduo 1 (left) and *hsp101–1* (right). **c**, **d** Comparison of seed setting and pollen fertility between wild type Huaduo 1 and *hsp101–1*. 2 L, 2S, 3 L, 3S, 4 L, 4S: leaf and stem at meiosis stage, bicellular pollen stage and 5 days after flowering; M, M1, M3: young panicle at the length of 0.5 cm 1 cm and 5 cm; PMAA, PMAO, MA1A, MA1O, MA2A, MA2O, SCPA, SCPO, SCP2A, SCP2O, BCPA, BCPO: anther and ovary at pre-meiotic interphase, meiosis I, meiosis II, microspore early, microspore late and bicellular pollen stages. Scale bars: 20 cm for plant, 1 cm for panicle and 100 μm for pollen

Other than seedling stage, *HSP101–1* was also presumed to play important role during reproductive stage. RNA-Seq data revealed that *HSP101–1* was highly expressed in anther at meiosis stage in neo-tetraploid rice Huaduo 1 under natural environment (Fig. S10a). Secondly, gene expression of *HSP101* in diploid rice, Nipponbare from RiceXPro database suggest that this gene was expressed in ovary at 1 day after flowering (Fig. S11). The difference in expression patterns indicated that this gene may play divergent functions in diploid and neo-tetraploid rice. qRT-PCR results further confirmed that *HSP101–1* was specifically expressed in anthers at PMA (pre-meiotic interphase), MA I (meiosis I) and MA II (meiosis II) under optimal conditions (without heat stress) (Fig. 6a). Knockout of *HSP101–1* in neo-tetraploid rice cause 24.99% reduction in seed setting and 31.78% reduction in pollen fertility (Fig. 6b, c and d). Cytological observation on pollen development of *hsp101–1* and wild type Huaduo 1 revealed degeneration of some microspores in tetrad stage (Fig. S13g) and asynchronous development of microspores in late microspore stage (Fig. S13l and S13p). These results revealed the function of *HSP101–1* in regulating fertility of neo-tetraploid rice. Moreover, the seed setting of late and early seasons, which have different temperatures at flowering stage, were compared in NTRs and ATRs. In the high temperature environment during early season (Fig. S10b), the seed set of ATRs were declined by 8.33% compared to late season, while the seed setting of NTRs increased by about 6.35% (Fig. S10c, Yu et al. 2020). These results indicated that *HSP101–1* not only regulate the thermotolerance ability at seedling stage, but also play important role during reproductive stages of NTRs.

## Discussion

### Construction of the First Large Scale Genomic Variation Repository of Tetraploid Rice

The high-quality genome sequencing and genomic variation data provided a way to precisely resolve the population structure of plants, which also facilitated the detection of functional genes in rice (Wang et al. 2018b), maize (Lai et al. 2010), soybean (Lam et al. 2011) and cotton (Ma et al. 2018). NTRs are important fertile materials for tetraploid rice breeding, and the development of NTRs provided rice breeders a new direction for the improvement and commercial utilization of tetraploid rice (Guo et al. 2017; Koide et al. 2020; Ghaleb et al. 2020). Understanding the genetic variations of NTRs will largely facilitate their utilization and gene functional analysis. In this context, the genomic variations of 15 NTRs and 18 ATRs were detected by using whole genome re-sequencing. This is the first genomic variation repository of tetraploid rice that will help us to find the responsible factors governing yield related traits in polyploid rice. Here, a total of 2 billion high quality pair-end sequencing reads of tetraploid rice were generated that covered the MSU7 and R498 reference genome by about 46.85-fold coverage depth. A total of 7,445,008 and 7,880,885 variations were detected between 33 tetraploid rice lines and reference genomes of MSU7 and R498, and the low-diversity region on Chr5 was overlapped with recently published genomes of four rice genotypes (Angad et al. 2020). These genomic variations will play an important role to decipher the underlying molecular mechanisms associated with yield performance and further improvement of tetraploid rice.

### Neo-Tetraploid Rice Was Classified as Independent *japonica* Subpopulation with Unique Variations

Population structure and subspecies classification are important characterizations of new crop germplasm, which helps breeders for their proper utilization. As a novel kind of tetraploid rice, there is no information about the classification of NTR. The results of PCA, ADMIXTURE and phylogenetic analysis classified tetraploid (including NTRs and ATRs) rice accessions into five subpopulations, which were consistent with their features of subspecies classification. Together with the *indica*/*japonica* reference genome coverage rate and genomic variations, NTRs can be classified into *japonica* subspecies with independent position. The population structure of tetraploid rice will be helpful for finding new directions of tetraploid rice improvement. As the high heterosis effect between two rice subspecies, *japonica* and *indica*, has been reported, the subspecies classification information might be useful to find the best combination with the highest heterosis (Huang et al. 2015). The results of the present classification explained that why NTRs produced high heterosis when crossed with *indica* tetraploid rice in a previous study (Guo et al. 2017). More importantly, wide-compatible gene, *S*_*5*_^*n*^, was also detected in NTRs in this study that was consistent with previous reports, which indicated the promising utilization of NTRs in construction of tetraploid rice hybrids, especially subspecies hybrids (Chen et al. 2008; Guo et al. 2017; Chen et al. 2019; Ghaleb et al. 2020). Although further studies are required to comprehensively reveal fertility mechanism, these findings partially explained that why hybrids between NTRs and ATRs have higher fertility than the hybrids generated form ATRs. The independent population position of NTRs also indicated the existence of NTR specific variations, which provides new information for rice genetic variation resource library and would be important for the improvement of tetraploid rice.

The detection of unique genomic variations that present in the genome of NTRs, but not in ATRs, was considered as the interesting results, which supported the theory of “genome shock” (McClintock 1984; Xu et al. 2014). The autotetraploid rice was developed from the genome doubling of diploid rice. However, few genomic variations were detected between autotetraploid rice and their diploid counterpart. This phenomenon has been reported by previous studies, for example, there were only 185 homozygous variations (78 SNPs and 107 InDels) between ATR 02428-4x and its diploid counterpart 02428-2x (Li et al. 2018); and only 81 SNPs and 182 InDels were identified in ATR, T449, compared to its diploid counterpart, E249 (Chen et al. 2018). These variations between ATRs and their diploid counterparts tend to be occurred randomly in genome, so there was no unique haplotype detected between ATRs and diploid rice. In allopolyploids, several findings of novel variations support the theory of “genome shock” (McClintock 1984). Novel genomic variations have been detected in the polyploids of Arabidopsis (Madlung et al. 2005), *Brassica napus* (Gaeta et al. 2007; Song et al. 1995) and wheat (Han et al. 2005; Ozkan et al. 2001; Shaked et al. 2001). Recently, enormous amount of homoeologous exchanges (HEs) were found in segmental allotetraploid rice, which responsible for their genomic and phenotypic variations (Wu et al. 2020). Here, NTRs were developed from the crossing decedents of ATRs from different ecotypes, which can be considered as a new segmental allotetraploid rice. The hypothesis is that the unique haplotypes in NTRs compared with DRs may occur during their breeding process of early generations, and the beneficial alleles were selected and fixed in subsequent generations.

### Novel Allele, *HSP101–1*, Is Indispensable for Thermotolerance and High Fertility in Neo-Tetraploid Rice

Climate change or global warming may aggravate the decrease in yield and quality of rice (Battisti and Naylor 2009; Welch et al. 2010). The genetically improved rice is considered important to alleviate the threats. *HSP101* is a member of HSP100/ClpB family, which is a crucial heat shock protein that regulates thermotolerance to cells, and over-expression of *HSP101* significantly improved the thermotolerance ability of diploid rice (Katiyar-Agarwal et al. 2003). The function of *HSP101* in heat tolerance was already reported (Hong and Vierling 2001), and its pleiotropic function in regulating development and fitness in plants was also found (Nieto-Sotelo et al. 2002; Tonsor et al. 2008). The accumulation of *HSP101* was found in mature maize kernels under optimal temperature and disappeared after 3 days of imbibition. Its negative influences were revealed between *HSP101* and growth rate of the primary root in addition to its function in thermotolerance (Nieto-Sotelo et al. 2002). *HSP101* may enhance plant fitness in normal growth conditions, since the loss of function of *HSP101* reduced about 33% fruits in *Arabidopsis thaliana* (Tonsor et al. 2008).

The identified novel allele of *HSP101–1* in neo-tetraploid rice was specific to all the high yielding neo-tetraploid rice lines, which was absent in autotetraploid rice, 3KRG and RiceVarMap2 database. Consistent with diploid rice, *HSP101–1* is a heat response factor at seedling stage in neo-tetraploid rice, and its association with NTR seedling heat tolerance was further proved by CRISPR/Cas9 gene knockout. *HSP101–1* also showed potential to improve fertility and yield in the high temperature environment of early season. Other than thermotolerance, *HSP101–1* also plays functional role in reproductive development. The expression of *HSP101–1* was detected in anthers at pre-meiotic (PMA), meiosis I and meiosis II stages of neo-tetraploid rice. Moreover, loss-of-function mutant of *HSP101–1* showed significant reduction in seed setting of neo-tetraploid rice. Hence, the novel allele of *HSP101–1* is indispensable for high fertility in neo-tetraploid rice. These results revealed enriched allelic diversity of *HSP101* gene and suggested promising potential function of this novel allele in heat stress and maintaining high fertility of neo-tetraploid rice.

## Conclusions

Large-scale genomic variations of autotetraploid rice and neo-tetraploid rice lines, and population structure analysis classified neo-tetraploid rice lines into *japonica* subspecies with independent subpopulation position. Haplotype patterns of whole genome genes, especially yield related key genes, were analyzed in tetraploid rice population. Functional validation indicated that the novel haplotype of *HSP101–1* plays important role in both seedling heat stress and fertility in neo-tetraploid rice. Here, the construction of first large-scale genomic repository of autotetraploid and neo-tetraploid rice, and the revelation of classification and population structure will play important role in optimizing the designs of tetraploid rice breeding. The specific variations in NTRs will facilitate further marker-assisted selection and functional genomics of tetraploid rice.

## Materials and Methods

### Calculation of Quantity and Quality of Genome Sequencing Data

Genome sequencing data of tetraploid rice, including 15 neo-tetraploid rice lines (NTRs) and 18 autotetraploid rice lines (ATRs) were collected from our bioproject PRJNA526117. The previously published diploid rice genome sequencing data of 38 accessions (8 common wild rice lines, 15 *indica* rice lines and 15 *japonica* rice lines) were downloaded from the NCBI SRA database with the bioproject accession number PRJNA396096 (Yu et al. 2018), PRJNA526117 (Yu et al. 2020) and PRJNA407820 (Wang et al. 2018a) using the Aspera Connect (version 3.7.4.147727) software, and the downloaded SRA files were transformed into fastq format using the fastq-dump command in SRAToolkit (http://ncbi.github.io/sra-tools/). The quantity and quality of raw sequencing data was evaluated using the FastQC (v0.11.6) software (Andrews 2010) and the low-quality sequencing reads were removed.

### Reads Mapping and Variation Calling

The sequencing reads that passed the quality control process were mapped onto the MSU7 (Nipponbare, *O. sativa japonica*) and R498 (Shuhui498, *O. sativa indica*) reference genome using BWA (0.7.17-r1188) (Li and Durbin 2009), and MarkDuplicates in Picard (Broad Institute 2019) were used to eliminate data of PCR duplication. The SAM files were sorted, indexed, and converted to BAM format using SAMtools (version 1.9) (Li 2011). The genomeCoverageBed in bedtools (v2.27.1) was used to estimate the reference genome coverage (Quinlan and Hall 2010). Genome Analysis Toolkit (GATK, version 3.8–0) was used to call the variations from the alignment file, and the analysis pipeline was constructed based on the GATK best practices (Mckenna et al. 2010). The variations were annotated by SnpEff (4.3 s) based on the annotation GFF3 files of two reference genomes (Cingolani et al. 2012), and variations were compared by using VCFtools (0.1.16) software (Danecek et al. 2011). Variation density of ATRs and NTRs was calculated using 100 Kb sliding windows, and the density distribution was illustrated using TBtools (Chen et al. 2020). In order to estimate the fraction of *japonica* and *indica* genomes present in the ATRs and NTRs, the variations between MSU7 and R498 genomes were detected by aligning the R498 to MSU7 genome. The generated SNPs and InDels were used as markers to calculate the fractions, and the results were illustrated by bar plots.

### Population Structure and Phylogeny Analysis

SNP variations were selected using “SelectVariants” from GATK (3.8–0) (Mckenna et al. 2010), and PCA (principal components analysis) was conducted using GCTA (1.91.4) software (Yang et al. 2011) based on the whole genome SNP variations and the result was plotted using a script based on ggplot2 (Wickham 2016). A python script (Ortiz 2019) was used to convert the SNP VCF file to PHYLIP format. The PHYLIP format was further converted to MEGA file using MEGA X (version 10.0.4) (Kumar et al. 2018). Phylogenetic neighbor-joining tree was constructed with the bootstrap test of 1000, and the tree was visualized using FigTree (v1.4.3) software (https://github.com/rambaut/figtree). The population structure was analyzed using ADMIXTURE (version 1.3.0) software (Alexander and Lange 2011) with *k* (the number of groups) ranged from 2 to 8, and the *k* = 5 was a sensible modeling choice with the lowest cross validation error. The ancestry distributions of individuals were visualized using R script.

### Phenol Reaction of NTR Grains

The phenol reaction method was used to validate the *indica*-*japonica* classification of 15 NTRs (Wang et al. 2012). Autotetraploid rice line Huanghuazhan-4x and its diploid counterpart, autotetraploid rice line Taichung65-4x and its diploid counterpart were used as *indica* and *japonica* controls. Fifty grains were randomly selected from each line and were soaked into 2% phenol solution for 48 h, and were washed by water. The color of glumes was observed and photos were taken of all dried seeds.

### Haplotype Detection, Validation, and Construction of Gene Phylogenetic Tree

To conduct haplotype analysis, the nonsynonymous variations of 15 NTRs and 18 ATRs were selected and combined into one file. Then, the variations (SNPs and InDels) were used to construct the haplotype by our python script. The haplotypes with homozygous variations were considered as main haplotypes, while the haplotypes with heterozygous bases were still been counted in the total haplotype numbers. Firstly, a total of 717 agronomically important genes for yield that previously reported (Yonemaru et al. 2010; Abbai et al. 2019) in diploid rice were summarized (Table S8). The haplotype patterns of those genes in tetraploid rice population were further analyzed and haplotype number was counted. Secondly, haplotype analysis of 55,801 genes that annotated in rice reference genome (MSU7) was conducted in 15 NTRs and 18 ATRs. The zero-diversity genes, which were conserved in all tetraploid rice lines, were identified and the GO enrichment analysis were conducted using the agriGO v2.0 (Tian et al. 2017). To construct gene phylogenetic tree, the gene consensus sequences in our lines were obtained using consensus command in BCFtools (Danecek et al. 2011) and the phylogenetic neighbor-joining was constructed with the bootstrap test of 1000 in MEGA X (version 10.0.4) (Kumar et al. 2018). Haplotype network of *HSP101* was constructed using the sequence of each sample by DnaSP6 (Rozas et al. 2017) and Network software (Bandelt et al. 1999). Gene structure was plotted using IBS (Liu et al. 2015). Primers were designed using Primer Blast software in NCBI, and the segment of neo-tetraploid and autotetraploid containing variations were amplified and the PCR products were sequenced using Sanger sequencing. The sequences were aligned using ClustalW Multiple Alignment in BioEdit software. The NTR specific variation in *HSP101–1* was checked in the RFGB (Wang et al. 2020) and RiceVarMap2 (Zhao et al. 2015) databases.

### Real-Time Quantitative Reverse Transcription PCR (qRT-PCR) Analysis

The seedlings of neo-tetraploid rice line, Huaduo 1, at 3-leaf stage was treated at 42 °C for 1 h and then seedlings were transferred to growth chamber at 28 °C for 1, 2 and 3 h. The leaf (L), leaf sheath (S) and root (R) after each hour were collected and kept in liquid nitrogen and stored at − 80 °C. The samples from untreated seedlings were also collected as CK, and all the samples were taken in three biological replications. Total RNA from each sample was extracted and the reverse transcription was conducted using Evo M-MLV RT Kit with gDNA Clean for qPCR (Accurate Biotechnology). Primers used for qRT-PCR were designed using Primer Blast software in NCBI. The reaction of reverse transcription was as follow: incubation for 2 min at 42 °C, followed by RT process of 37 °C for 15 min, 85 °C for 5 s and then stored at 4 °C. The qRT-PCR was performed on Lightcycler480 (Roche) using the Hieff qPCR SYBR Green Master Mix (YEASEN). The reaction conditions were as follow: 30 s at 95 °C, 40 cycles of 95 °C denaturation for 5 s and 58 °C annealing and extension for 20s. Three biological replications were performed in this experiment. Actin was used as an internal reference for qRT-PCR. The relative gene expression was calculated by the 2^*–ΔΔCT*^ method (Livak and Schmittgen 2001).

### Construction of CRISPR/Cas9 Vector and Characterization of Gene Knockout Mutants

The targetDesign tool of CRISPR-GE was used to design two target-site primers for NTR specific gene, *HSP101–1*, using its genomic DNA sequence (http://skl.scau.edu.cn/home/) (Table S14, Xie et al. 2017). The constructed CRISPR/Cas9 vector (Butt et al. 2019; Ma et al. 2019) was transferred into NTR, Huaduo 1. Mutations were validated by Sanger sequencing using gene-specific target site primer. Sanger sequencing data were decoded using DSDecodeM program of CRISPR-GE tool kit (Fig. S12, Table S15, Xie et al. 2017). The thermotolerance of *hsp101–1* was checked in T1 generation by thermotolerance assay (see below). Seed setting of 16 T1 plants were investigated and compared with wild type, Huaduo 1. Pollen fertility was observed by using 1% I_2_-KI method with Motic BA200 microscope (Ghouri et al. 2019). Pollen development was observed by a whole mount eosin B confocal laser scanning microscopy (WE-CLSM) according to Wu et al. (2014).

### Heat Treatment and Thermotolerance Assays

Heat treatment of two neo-tetraploid rice (NTR) lines, Huaduo 1 and Huaduo 2543, were performed based on the previously established protocol for diploid rice (Lin et al. 2014) with minor modifications. *Indica* autotetraploid Huanghuazhan-4x and its diploid counterpart, *japonica* autotetraploid Taichung65-4x and its diploid counterpart and the parental lines of NTR were used as controls. To test basal thermo-tolerance ability, three-day-old seedlings that grown in an incubator at 28 °C in water were treated at 48 °C for 1 h, and then the seedlings were transferred to growth chamber at 28 °C for about 72 h. After that, the root and seedling lengths of treated and untreated samples (CK) were measured and their morphology was photographed. The index of heat sensitivity was calculated based on the decrease in seedling length compared to the untreated samples.

### Expression Pattern Analysis of *HSP101–1* in Neo-Tetraploid Rice

The expression information of *HSP101–1* was firstly predicted using our transcriptome data of Huaduo 1 (Yu et al. 2020), and expression pattern was illustrated using pheatmap (Kolde, 2019). Secondly, the expression pattern of *HSP101–1* was validated by qRT-PCR (see above). A total of 21 samples, including leaf and stem at meiosis stage, bicellular pollen stage and 5 days after flowering, young panicle at the length of 0.5 cm, 1 cm and 5 cm, anther and ovary at meiosis I, meiosis II, microspore early, microspore late and bicellular pollen stages, were used to illustrate the expression pattern of *HSP101–1* in neo-tetraploid rice Huaduo 1. The expression pattern of *HSP101* in Nipponbare were retrieved from RiceXPro database (Sato et al. 2010).

## Supplementary Information

**Additional file 1: Figure S1.** Circos plot illustrating the variation (including SNPs and InDels) density of different types of tetraploid rice against MSU7 reference genome. **Figure S2.** Circos plot illustrating the variation (including SNPs and InDels) density of different types of tetraploid rice against R498 reference genome. **Figure S3.** Difference of 5-fold mapping coverage ratio between *japonica* reference genome MSU7 and *indica* reference genome R498. **Figure S4.** Comparison of genomic variation counts that generated based on japonica reference genome MSU7 and indica reference genome R498. **Figure S5.** Phenol reaction of neo-tetraploid rice lines. **Figure S6.** Sequencing coverage of wide compatibility gene *S5* (*LOC_Os06g11010*) in autotetraploid rice lines and neo-tetraploid rice lines. **Figure S7.** Distribution of haplotypes per gene of the 55,801 annotated genes in tetraploid rice. **Figure S8.** Distribution of haplotypes per gene in the 717 agronomically important genes in tetraploid rice. **Figure S9.** The phylogenetic tree of *LOC_Os04g09310* (a), *LOC_Os05g02840* (b), *LOC_Os05g43000* (c) and *LOC_Os10g25730* (d) in autotetraploid rice and neo-tetraploid rice lines. **Figure S10.** The expression pattern analysis of *HSP101* and the impact of different temperatures during flowering to the seed setting in tetraploid rice lines. **Figure S11.** The expression pattern analysis of HSP101 in diploid rice Nipponbare using the RiceXPro database. **Figure S12.** Sanger sequencing chromatogram of *hsp101–1* mutants. **Figure S13.** Cytological observation of pollen development of *hsp101–1* and wild type Huaduo 1.

**Additional file 2: Table S1.** Summary of quantity and quality of genome re-sequencing data. **Table S2.** Genome coverage of sequencing reads to MSU7 and R498 reference genome. **Table S3.** Number of genomic variations against MSU7 and R498 reference genome. **Table S4.** Number of genomic variations from different effect levels against MSU7 reference genome. **Table S5.** Variation density of different types of autotetraploid rice against MSU7 and R498 reference genome. **Table S6.** Cross-validation error of different *k* values in ADMIXTURE analysis. **Table S7.** GO enrichment analysis of zero-diversity genes in tetraploid rice population. **Table S8.** Annotation of 717 agronomically important genes that previously reported in diploid rice. **Table S9.** Haplotype patterns of 717 agronomically important genes in autotetraploid rice (ATR) and neo-tetraploid rice (NTR) lines. **Table S10.** Neo-tetraploid rice (NTR) specific haplotypes compared with 30 diploid rice cultivars. **Table S11.** Validation of neo-tetraploid rice specific variation in *HSP101–1* using Sanger sequencing. **Table S12.** The genomic variations in 3024 diploid rice accessions of *HSP101* from rice functional genomics and breeding (RFGB) database. **Table S13.** The genomic variations in diploid rice accessions of *HSP101* from RiceVarMap2 database. **Table S14.** Two target-site primers for NTR specific gene, *HSP101–1*. **Table S15.** Mutations of *HSP101–1* knockout plants.

## Data Availability

The raw reads of whole-genome resequencing of autotetraploid rice and neo-tetraploid rice were available at the NCBI Sequence Read Archive with accession ID PRJNA526117. The downloaded sequencing data of 38 varieties are available from NCBI SRA database with the bioproject accessions are PRJNA396096 and PRJNA407820. The sequences and annotations of rice *japonica* reference genome MSU7 and *indica* reference genome R498 is available from the websites http://rice.plantbiology.msu.edu/ and http://www.mbkbase.org/R498/, respectively.

## Abbreviations

NTR: Neo-tetraploid rice
ATR: Autotetraploid rice
DR: Diploid rice
WGD: Whole-genome duplication
GO: Gene ontology
SNP: Single-nucleotide polymorphisms
PCA: Principal component analysis
PMA: Pre-meiotic interphase
MAI: Meiosis I
MAII: Meiosis II
qRT-PCR: Real-time quantitative reverse transcription PCR

## References

[CR1] Abbai R, Singh VK, Nachimuthu VV, Singh VK, Nachimuthu VV, Sinha P, Selvaraj R, Vipparla AK, Singh AK, Singh UM, Varshney RK, Kumar A (2019). Haplotype analysis of key genes governing grain yield and quality traits across 3KRG panel reveals scope for the development of tailor-made rice with enhanced genetic gains. Plant Biotechnol J.

[CR2] Alexander DH, Lange K (2011). Enhancements to the ADMIXTURE algorithm for individual ancestry estimation. BMC Bioinformatics.

[CR3] Andrews S (2010). FastQC: a quality control tool for high throughput sequence data.

[CR4] Angad K, Anurag D, Arvind K, Vinay K, S Gopala K, Subhasish M, Bhaskar CP, Ashok KS, Akhilesh KT, Swarup KP, Jitendra KT (2020). Genome-wide analysis of polymorphisms identified domestication-associated long low-diversity region carrying important rice grain size/weight quantitative trait loci. Plant J.

[CR5] Bandelt H-J, Forster P, Röhl A (1999). Median-joining networks for inferring intraspecific phylogenies. Mol Biol Evol.

[CR6] Battisti DS, Naylor RL (2009). Historical warnings of future food insecurity with unprecedented seasonal heat. Science.

[CR7] Bei XJ, Shahid MQ, Wu JW, Chen ZX, Wang L, Liu XD (2019). Re-sequencing and transcriptome analysis reveal rich DNA variations and differential expressions of fertility-related genes in neo-tetraploid rice. PLoS One.

[CR8] Blanc G, Wolfe KH (2004). Widespread paleopolyploidy in model plant species inferred from age distributions of duplicate genes. Plant Cell.

[CR9] Broad Institute (2019). A set of command line tools (in Java) for manipulating high-throughput sequencing (HTS) data and formats.

[CR10] Butt H, Eid A, Momin AA, Bazin J, Crespi M, Arold ST, Mahfouz MM (2019). CRISPR directed evolution of the spliceosome for resistance to splicing inhibitors. Genome Biol.

[CR11] Cai DT, Chen JG, Chen DL, Dai BC, Zhang W, Song ZJ, Yang ZF, Du CQ, Tang ZQ, He YC, Zhang DS, He GC, Zhu YG (2007). The breeding of two polyploid rice lines with the characteristic of polyploid meiosis stability. Sci China Ser C-Life Sci.

[CR12] Cavanagh CR, Chao S, Wang S, Huang BE, Stephen S, Kiani S, Forrest K, Saintenac C, Brown-Guedira GL, Akhunova A, See D, Bai G, Pumphrey M, Tomar L, Wong D, Kong S, Reynolds M, Da Silva ML, Bockelman H, Talbert L, Anderson JA, Dreisigacker S, Baenziger S, Carter A, Korzun V, Morrell PL, Dubcovsky J, Morell MK, Sorrells ME, Hayden MJ, Akhunov E (2013). Genome-wide comparative diversity uncovers multiple targets of selection for improvement in hexaploid wheat landraces and cultivars. Proc Natl Acad Sci U S A.

[CR13] Chao S, Dubcovsky J, Dvorak J, Luo MC, Baenziger SP, Matnyazov R, Clark DR, Talbert LE, Anderson JA, Dreisigacker S, Glover K, Chen J, Campbell K, Bruckner PL, Rudd JC, Haley S, Carver BF, Perry S, Sorrells ME, Akhunov ED (2010). Population- and genome-specific patterns of linkage disequilibrium and SNP variation in spring and winter wheat (*Triticum aestivum* L.). BMC Genomics.

[CR14] Chen CJ, Chen H, Zhang Y, Thomas HR, Frank MH, He YH, Xia R (2020). TBtools: an integrative toolkit developed for interactive analyses of big biological data. Mol Plant.

[CR15] Chen JJ, Ding JH, Ouyang YD, Du HY, Yang JY, Cheng K, Zhao J, Qiu SQ, Zhang XL, Yao JL, Liu KD, Wang L, Xu CG, Li XH, Xue YB, Xia M, Ji Q, Lu JF, Xu ML, Zhang QF (2008). A triallelic system of *S5* is a major regulator of the reproductive barrier and compatibility of *indica*-*japonica* hybrids in rice. Proc Natl Acad Sci U S A.

[CR16] Chen L, Shahid MQ, Wu JW, Chen ZX, Wang L, Liu XD (2018). Cytological and transcriptome analyses reveal abrupt gene expression for meiosis and saccharide metabolisms that associated with pollen abortion in autotetraploid rice. Mol Gen Genomics.

[CR17] Chen L, Yuan Y, Wu JW, Chen ZX, Wang L, Shahid MQ, Liu XD (2019). Carbohydrate metabolism and fertility related genes high expression levels promote heterosis in autotetraploid rice harboring double neutral genes. Rice.

[CR18] Cingolani P, Platts A, Wang LL, Coon M, Nguyen T, Wang L, Land SJ, Lu X, Ruden DM (2012). A program for annotating and predicting the effects of single nucleotide polymorphisms, SnpEff: SNPs in the genome of Drosophila melanogaster strain w^1118^; iso-2; iso-3. Fly (Austin).

[CR19] Danecek P, Auton A, Abecasis G, Albers CA, Banks E, MA DP, Handsaker RE, Lunter G, Marth GT, Sherry ST, McVean G, Durbin R, 1000 Genomes Project Analysis Group (2011). The variant call format and VCFtools. Bioinformatics.

[CR20] De Leon TB, Karn E, Al Khatib K, Espino L, Blank T, Andaya CB, Andaya VC, Brim DeForest W (2019). Genetic variation and possible origins of weedy rice found in California. Ecol Evol.

[CR21] Dixit N, Dokku P, Amitha Mithra SV, Parida SK, Singh AK, Singh NK, Mohapatra T (2013). Haplotype structure in grain weight gene *GW2* and its association with grain characteristics in rice. Euphytica.

[CR22] Duitama J, Silva A, Sanabria Y, Cruz DF, Quintero C, Ballen C, Lorieux M, Scheffler B, Farmer A, Torres E, Oard J, Tohme J (2015). Whole genome sequencing of elite rice cultivars as a comprehensive information resource for marker assisted selection. PLoS One.

[CR23] Gaeta RT, Pires JC, Iniguez-Luy F, Leon E, Osborn TC (2007). Genomic changes in resynthesized Brassica napus and their effect on gene expression and phenotype. Plant Cell.

[CR24] Ghaleb MAA, Li C, Shahid MQ, Yu H, Liang JH, Chen RX, Wu JW, Liu XD (2020). Heterosis analysis and underlying molecular regulatory mechanism in a wide-compatible neo-tetraploid rice line with long panicles. BMC Plant Biol.

[CR25] Ghouri F, Zhu J, Yu H, Wu J, Baloch FS, Liu X, Shahid MQ (2019). Deciphering global DNA variations and embryo sac fertility in autotetraploid rice line. Turk J Agric For.

[CR26] Guo H, Mendrikahy JN, Xie L, Deng JF, Lu ZJ, Wu JW, Li X, Shahid MQ, Liu XD (2017) Transcriptome analysis of neo-tetraploid rice reveals specific differential gene expressions associated with fertility and heterosis. Sci Rep 7:40139. 10.1038/srep40139. 10.1038/srep40139 PMC5223177 28071676

[CR27] Guo HB, Liu XD (2014). The research on autotetraploid rice.

[CR28] Guo HB, Shahid MQ, Zhao J, Li YJ, Wang L, Liu XD (2016). Agronomic traits and cytogenetic evaluation of newly developed autotetraploid rice line. Pak J Agric Sci.

[CR29] Han F, Fedak G, Guo W, Liu B (2005). Rapid and repeatable elimination of a parental genome-specific DNA repeat (pGc1R-1a) in newly synthesized wheat allopolyploids. Genetics.

[CR30] He JH, Shahid MQ, Chen ZX, Chen XA, Liu XD (2011). Abnormal PMC microtubule distribution pattern and chromosome behavior resulted in low pollen fertility of an intersubspecific autotetraploid rice hybrid. Plant Syst Evol.

[CR31] Hong SW, Vierling E (2001). Hsp101 is necessary for heat tolerance but dispensable for development and germination in the absence of stress. Plant J.

[CR32] Huang X, Yang S, Gong J, Zhao Y, Feng Q, Gong H, Li W, Zhan Q, Cheng B, Xia J, Chen N, Hao Z, Liu K, Zhu C, Huang T, Zhao Q, Zhang L, Fan D, Zhou C, Lu Y, Weng Q, Wang ZX, Li J, Han B (2015). Genomic analysis of hybrid rice varieties reveals numerous superior alleles that contribute to heterosis. Nat Commun.

[CR33] Katiyar-Agarwal S, Agarwal M, Grover A (2003). Heat-tolerant basmati rice engineered by over-expression of *hsp101*. Plant Mol Biol.

[CR34] Koide Y, Kuniyoshi D, Kishima Y (2020). Fertile Tetraploids: new resources for future Rice breeding?. Front Plant Sci.

[CR35] Kolde R (2019). Pheatmap: Pretty Heatmaps. R package version 1.0.12. https://CRAN.R-project.org/package=pheatmap

[CR36] Kumar S, Stecher G, Li M, Knyaz C, Tamura K (2018). MEGA X: molecular evolutionary genetics analysis across computing platforms. Mol Biol Evol.

[CR37] Lai JS, Li RQ, Xu X, Jin WW, Xu ML, Zhao HN, Xiang ZK, Song WB, Ying K, Zhang M, Jiao YP, Ni PX, Zhang JG, Li D, Guo XS, Ye KX, Jian M, Wang B, Zheng HS, Liang HQ, Zhang XQ, Wang SC, Chen SJ, Li JS, Fu Y, Nathan MS, Yang HM, Wang J, Dai JR, Patrick SS, Wang J (2010). Genome-wide patterns of genetic variation among elite maize inbred lines. Nat Genet.

[CR38] Lam HM, Xu X, Liu X, Chen WB, Yang GH, Wong FL, Li MW, He WM, Qin N, Wang B, Li J, Jian M, Wang J, Shao GH, Wang J, Sun SSM, Zhang GY (2011). Resequencing of 31 wild and cultivated soybean genomes identifies patterns of genetic diversity and selection. Nat Genet.

[CR39] Li H (2011). A statistical framework for SNP calling, mutation discovery, association mapping and population genetical parameter estimation from sequencing data. Bioinformatics.

[CR40] Li H, Durbin R (2009). Fast and accurate short read alignment with burrows-wheeler transform. Bioinformatics.

[CR41] Li X, Shahid MQ, Xia J, Lu ZJ, Fang N, Wang L, Wu JW, Chen ZX, Liu XD (2017) Analysis of small RNAs revealed differential expressions during pollen and embryo sac development in autotetraploid rice. BMC Genomics 18(1):129. 10.1186/s12864-017-3526-8 10.1186/s12864-017-3526-8 PMC5295217 28166742

[CR42] Li X, Yu H, Jiao YM, Shahid MQ, Wu JW, Liu XD (2018). Genome-wide analysis of DNA polymorphisms, the methylome and transcriptome revealed that multiple factors are associated with low pollen fertility in autotetraploid rice. PLoS One.

[CR43] Lin MY, Chai KH, Ko SS, Kuang LY, Lur HS, Charng YY (2014). A positive feedback loop between HEAT SHOCK PROTEIN 101 and HEAT STRESS-ASSOCIATED 32-KD PROTEIN modulates long-term acquired thermotolerance illustrating diverse heat stress responses in rice varieties. Plant Physiol.

[CR44] Liu WZ, Xie YB, Ma JY, Luo XT, Nie P, Zuo ZX, Lahrmann U, Zhao Q, Zheng YY, Zhao Y, Xue Y, Ren J (2015). IBS: an illustrator for the presentation and visualization of biological sequences. Bioinformatics.

[CR45] Livak KJ, Schmittgen TD (2001). Analysis of relative gene expression data using real-time quantitative PCR and the 2^–ΔΔCT^ method. Methods.

[CR46] Lu Z, Guo X, Huang Z, Xia J, Li X, Wu J, Yu H, Shahid MQ, Liu X (2020). Transcriptome and gene editing analyses reveal *MOF1a* defect alters the expression of genes associated with tapetum development and chromosome behavior at meiosis stage resulting in low pollen fertility of tetraploid rice. Int J Mol Sci.

[CR47] Luan L, Tu SB, Long WB, Wang X, Liu YH, Kong FL, He T, Yan WG, Yu MQ (2007). Cytogenetic studies on two F_1_ hybrids of autotetraploid rice varieties showing extremely high level of heterosis. Plant Syst Evol.

[CR48] Ma K, Han JL, Yao Y, Yang ZF, Chen JY, Liu YG, Zhu QL, Chen LT (2019). An effective strategy to establish a male sterility mutant mini-library by CRISPR/Cas9-mediated knockout of anther-specific genes in rice. J Genet Genomics.

[CR49] Ma ZY, He SP, Wang XF, Sun JL, Zhang Y, Zhang GY, Wu LQ, Li ZK, Liu ZH, Sun GF, Yan YY, Jia YH, Yang J, Pan ZE, Gu QS, Li XY, Sun ZW, Dai PH, Liu ZW, Gong WF, Wu JH, Wang M, Liu HW, Feng KY, Ke HF, Wang JD, Lan HY, Wang GN, Peng J, Wang N, Wang LR, Pang BY, Peng Z, Li RQ, Tian SL, Du XM (2018). Resequencing a core collection of upland cotton identifies genomic variation and loci influencing fiber quality and yield. Nat Genet.

[CR50] Maccaferri M, Sanguineti MC, Noli E, Tuberosa R (2005). Population structure and long-range linkage disequilibrium in a durum wheat elite collection. Mol Breed.

[CR51] Madlung A, Tyagi AP, Watson B, Jiang H, Kagochi T, Doerge RW, Martienssen R, Comai L (2005). Genomic changes in synthetic Arabidopsis polyploids. Plant J.

[CR52] Malysheva-Otto LV, Ganal MW, Röder MS (2006). Analysis of molecular diversity, population structure and linkage disequilibrium in a worldwide survey of cultivated barley germplasm (*Hordeum vulgare* L.). BMC Genet.

[CR53] Masterston J (1994). Stomatal size in fossil plants: evidence for polyploidy in majority of angiosperms. Science.

[CR54] McClintock B (1984). The significance of responses of the genome to challenge. Science.

[CR55] Mckenna A, Hanna M, Banks E, Sivachenko A, Cibulskis K, Kernytsky A, Garimella K, Altshuler D, Gabriel S, Daly M, DePristo MA (2010). The genome analysis toolkit: a MapReduce framework for analyzing next-generation DNA sequencing data. Genome Res.

[CR56] Mishra S, Singh B, Misra P, Rai V, Singh NK (2016). Haplotype distribution and association of candidate genes with salt tolerance in Indian wild rice germplasm. Plant Cell Rep.

[CR57] Morris GP, Ramu P, Deshpande SP, Hash CT, Shah T, Upadhyaya HD, Riera-Lizarazu O, Brown PJ, Acharya CB, Mitchell SE, Harriman J, Glaubitz JC, Buckler ES, Kresovich S (2013). Population genomic and genome-wide association studies of agroclimatic traits in sorghum. Proc Natl Acad Sci U S A.

[CR58] Nieto-Sotelo J, Martínez LM, Ponce G, Cassab GI, Alagón A, Meeley RB, Ribaut J, Yang R (2002). Maize HSP101 plays important roles in both induced and basal thermotolerance and primary root growth. Plant Cell.

[CR59] Ortiz EM (2019). vcf2phylip v2.0: convert a VCF matrix into several matrix formats for phylogenetic analysis.

[CR60] Ozkan H, Levy AA, Feldman M (2001). Allopolyploidy-induced rapid genome evolution in the wheat (*Aegilops-Triticum*) group. Plant Cell.

[CR61] Quinlan AR, Hall IM (2010). Bedtools: a flexible suite of utilities for comparing genomic features. Bioinformatics.

[CR62] Renny-Byfield S, Wendel JF (2014). Doubling down on genomes: polyploidy and crop plants. Am J Bot.

[CR63] Romay MC, Millard MJ, Glaubitz JC, Peiffer JA, Swarts KL, Casstevens TM, Elshire RJ, Acharya CB, Mitchell SE, Flint-Garcia SA, McMullen MD, Holland JB, Buckler ES, Gardner CA (2013). Comprehensive genotyping of the USA national maize inbred seed bank. Genome Biol.

[CR64] Rozas J, Ferrer-Mata A, Sánchez-DelBarrio JC, Guirao-Rico S, Librado P, Ramos-Onsins SE, Sánchez-Gracia A (2017). DnaSP 6: DNA sequence polymorphism analysis of large data sets. Mol Biol Evol.

[CR65] Sato Y, Antonio B, Namiki N, Takehisa H, Minami H, Kamatsuki K, Sugimoto K, Shimizu Y, Hirochika H, Nagamura Y (2010). RiceXPro: a platform for monitoring gene expression in japonica rice grown under natural field conditions. Nucleic Acids Res.

[CR66] Serba DD, Muleta KT, St. Amand P, Bernardo A, Bai G, Perumal R, Bashir E (2019). Genetic diversity, population structure, and linkage disequilibrium of pearl millet. Plant Genome.

[CR67] Shahid MQ, Li YJ, Saleem MF, Wei CM, Naeem M, Liu XD (2013). Yield and yield components in autotetraploid and diploid rice genotypes (*indica* and *japonica*) sown in early and late seasons. Aust J Crop Sci.

[CR68] Shahid MQ, Liu GF, Li JQ, Muhammad N, Liu XD (2011). Heterosis and gene action study of agronomic traits in diploid and autotetraploid rice. Acta Agriculturae Scandinavica Sect B-Soil Plant Sci.

[CR69] Shahid MQ, Sun JF, Wei CM, Peng Z, Liu XD (2010). Study on the abnormality of embryo sac and pollen fertility in autotetraploid rice during different growing seasons. Pak J Bot.

[CR70] Shahid MQ, Xu HM, Lin SQ, Chen ZX, Muhammad N, Li YJ, Liu XD (2012). Genetic analysis and hybrid vigor study of grain yield and other quantitative traits in autotetraploid rice. Pak J Bot.

[CR71] Shaked H, Kashkush K, Ozkan H, Feldman M, Levy AA (2001). Sequence elimination and cytosine methylation are rapid and reproducible responses of the genome to wide hybridization and allopolyploidy in wheat. Plant Cell.

[CR72] Singh A, Singh PK, Singh R, Pandit A, Mahato AK, Gupta DK, Tyagi K, Singh AK, Singh NK, Sharma TR (2010). SNP haplotypes of the *BADH1* gene and their association with aroma in rice (*Oryza sativa* L.). Mol Breed.

[CR73] Song K, Lu P, Tang K, Osborn TC (1995) Rapid genome change in synthetic polyploids of brassica and its implications for polyploid evolution. Proc Natl Acad Sci U S A 92(17):7719-7723 10.1073/pnas.92.17.7719 PMC41217 7644483

[CR74] Tian T, Liu Y, Yan HY, You Q, Yi X, Du Z, Xu WY, Zhen S (2017). agriGO v2.0: a GO analysis toolkit for the agricultural community, 2017 update. Nucleic Acids Res.

[CR75] Tonsor SJ, Scott C, Boumaza I, Liss TR, Brodsky JL, Vierling E (2008). Heat shock protein 101 effects in *A. thaliana*: genetic variation, fitness and pleiotropy in controlled temperature conditions. Mol Ecol.

[CR76] Tu SB, Kong FL, Xu QF, He T (2003). Study on new system of heterosis utilization in autotetraploid rice. Bull Chin Acad Sci.

[CR77] Tu Y, Jiang AM, Gan L, Mokter H, Zhang JM, Peng B, Xiong YG, Song ZJ, Cai DT, Xu WF, Zhang JH, He YC (2014) Genome duplication improves rice root resistance to salt stress. Rice 7(1):15. 10.1186/s12284-014-0015-4 10.1186/s12284-014-0015-4 PMC4151024 25184027

[CR78] Van de Peer Y, Maere S, Meyer A (2009). The evolutionary significance of ancient genome duplications. Nat Rev Genet.

[CR79] Wang C, Yu H, Huang J, Wang W, Faruquee M, Zhang F, Zhao X, Fu B, Chen K, Zhang H, Tai S, Wei C, McNally KL, Alexandrov N, Gao X, Li J, Li Z, Xu J, Zheng T (2020). Towards a deeper haplotype mining of complex traits in rice with RFGB v2.0. Plant Biotechnol J.

[CR80] Wang CH, Xu Q, Yu P, Yuan XP, Yu HH, Wang YP, Tang SX, Wei XH (2012). Comparison of Cheng's index- and SSR markers-based classification of Asian cultivated rice. Ric Sci.

[CR81] Wang M, Li WZ, Fang C, Xu F, Liu YC, Wang Z, Yang R, Zhang M, Liu SL, Lu SJ, Lin T, Tang JY, Wang YQ, Wang HR, Lin H, Zhu BG, Chen MS, Kong FJ, Liu BH, Zeng DL, Jackson SA, Chu CC, Tian ZX (2018). Parallel selection on a dormancy gene during domestication of crops from multiple families. Nat Genet.

[CR82] Wang W, Mauleon R, Hu Z, Chebotarov D, Tai S, Wu Z, Li M, Zheng T, Fuentes RR, Zhang F, Mansueto L, Copetti D, Sanciangco M, Palis KC, Xu J, Sun C, Fu B, Zhang H, Gao Y, Zhao X, Shen F, Cui X, Yu H, Li Z, Chen M, Detras J, Zhou Y, Zhang X, Zhao Y, Kudrna D, Wang C, Li R, Jia B, Lu J, He X, Dong Z, Xu J, Li Y, Wang M, Shi J, Li J, Zhang D, Lee S, Hu W, Poliakov A, Dubchak I, Ulat VJ, Borja FN, Mendoza JR, Ali J, Li J, Gao Q, Niu Y, Yue Z, Naredo MEB, Talag J, Wang X, Li J, Fang X, Yin Y, Glaszmann J, Zhang J, Li J, Hamilton RS, Wing RA, Ruan J, Zhang G, Wei C, Alexandrov N, McNally KL, Li Z, Leung H (2018). Genomic variation in 3,010 diverse accessions of Asian cultivated rice. Nature.

[CR83] Welch JR, Vincent JR, Auffhammer M, Moya PF, Dobermann A, Dawe D (2010). Rice yields in tropical/subtropical Asia exhibit large but opposing sensitivities to minimum and maximum temperatures. Proc Natl Acad Sci U S A.

[CR84] Wickham H (2016). ggplot2: elegant graphics for data analysis.

[CR85] Wu JW, Hu CY, Shahid MQ, Guo HB, Zeng YX, Liu XD, Lu YG (2013) Analysis on genetic diversification and heterosis in autotetraploid rice. SpringerPlus 2:439. 10.1186/2193-1801-2-439 10.1186/2193-1801-2-439 PMC3773102 24046812

[CR86] Wu JW, Shahid MQ, Chen L, Chen ZX, Wang L, Liu XD, Lu YG (2015). Polyploidy enhances F_1_ pollen sterility loci interactions that increase meiosis abnormalities and pollen sterility in autotetraploid rice. Plant Physiol.

[CR87] Wu JW, Shahid MQ, Guo HB, Yin W, Chen ZX, Wang L, Liu XD, Lu YG (2014). Comparative cytological and transcriptomic analysis of pollen development in autotetraploid and diploid rice. Plant Reprod.

[CR88] Wu Y, Lin F, Zhou Y, Wang J, Sun S, Wang B, Zhang Z, Li G, Lin X, Wang X, Sun Y, Dong Q, Xu C, Gong L, Wendel JF, Zhang Z, Liu B (2020) Genomic mosaicism due to homoeologous exchange generates extensive phenotypic diversity in nascent allopolyploids. Natl Sci Rev nwaa277. 10.1093/nsr/nwaa277 10.1093/nsr/nwaa277 PMC8288387 34691642

[CR89] Xie XR, Ma XL, Zhu QL, Zeng DC, Li GS, Liu YG (2017). CRISPR-GE: a convenient software toolkit for CRISPR-based genome editing. Mol Plant.

[CR90] Xu C, Bai Y, Lin X, Zhao N, Hu L, Gong Z, Wendel JF, Liu B (2014). Genome-wide disruption of gene expression in allopolyploids but not hybrids of rice subspecies. Mol Biol Evol.

[CR91] Yang J, Lee SH, Goddard ME, Visscher PM (2011). GCTA: a tool for genome-wide complex trait analysis. Am J Hum Genet.

[CR92] Yang PM, Huang QC, Qin GY, Zhao SP, Zhou JG (2014). Different drought-stress responses in photosynthesis and reactive oxygen metabolism between autotetraploid and diploid rice. Photosynthetica.

[CR93] Yonemaru J, Yamamoto T, Fukuoka S, Uga Y, Hori K, Yano M (2010). Q-TARO: QTL annotation rice online database. Rice.

[CR94] Yu H, Shahid MQ, Li QH, Li YD, Li C, Lu ZJ, Wu JW, Zhang ZM, Liu XD (2020). Production assessment and genome comparison revealed high yield potential and novel specific alleles associated with fertility and yield in neo-tetraploid rice. Rice.

[CR95] Yu H, Shahid MQ, Li RB, Li W, Liu W, Fozia G, Liu XD (2018). Genome-wide analysis of genetic variations and the detection of rich variants of NBS-LRR encoding genes in common wild rice lines. Plant Mol Biol Report.

[CR96] Zhao H, Yao W, Ouyang Y, Yang W, Wang G, Lian X, Xing Y, Chen L, Xie W (2015). RiceVarMap: a comprehensive database of rice genomic variations. Nucleic Acids Res.

