# Supplementary material for: Genomics Analyses Reveal Unique Classification, Population Structure and Novel Allele of Neo-Tetraploid Rice
Source: Rice (N Y). 2021 Feb 6;14:16. doi: 10.1186/s12284-021-00459-y (PMC7867503; doi:10.1186/s12284-021-00459-y)
Supplement: Supplementary file 1 — Additional file 1: Figure S1. Circos plot illustrating the variation (including SNPs and InDels) density of different types of tetraploid rice against MSU7 reference genome. Figure S2. Circos plot illustrating the variation (including SNPs and InDels) density of different types of tetraploid rice against R498 reference genome. Figure S3. Difference of 5-fold mapping coverage ratio between japonica reference genome MSU7 and indica reference genome R498. Figure S4. Comparison of genomic variation counts that generated based on japonica reference genome MSU7 and indica reference genome R498. Figure S5. Phenol reaction of neo-tetraploid rice lines. Figure S6. Sequencing coverage of wide compatibility gene S5 (LOC_Os06g11010) in autotetraploid rice lines and neo-tetraploid rice lines. Figure S7. Distribution of haplotypes per gene of the 55,801 annotated genes in tetraploid rice. Figure S8. Distribution of haplotypes per gene in the 717 agronomically important genes in tetraploid rice. Figure S9. The phylogenetic tree of LOC_Os04g09310 (a), LOC_Os05g02840 (b), LOC_Os05g43000 (c) and LOC_Os10g25730 (d) in autotetraploid rice and neo-tetraploid rice lines. Figure S10. The expression pattern analysis of HSP101 and the impact of different temperatures during flowering to the seed setting in tetraploid rice lines. Figure S11. The expression pattern analysis of HSP101 in diploid rice Nipponbare using the RiceXPro database. Figure S12. Sanger sequencing chromatogram of hsp101–1 mutants. Figure S13. Cytological observation of pollen development of hsp101–1 and wild type Huaduo 1. [file 12284_2021_459_MOESM1_ESM.pdf]

Additional file 1

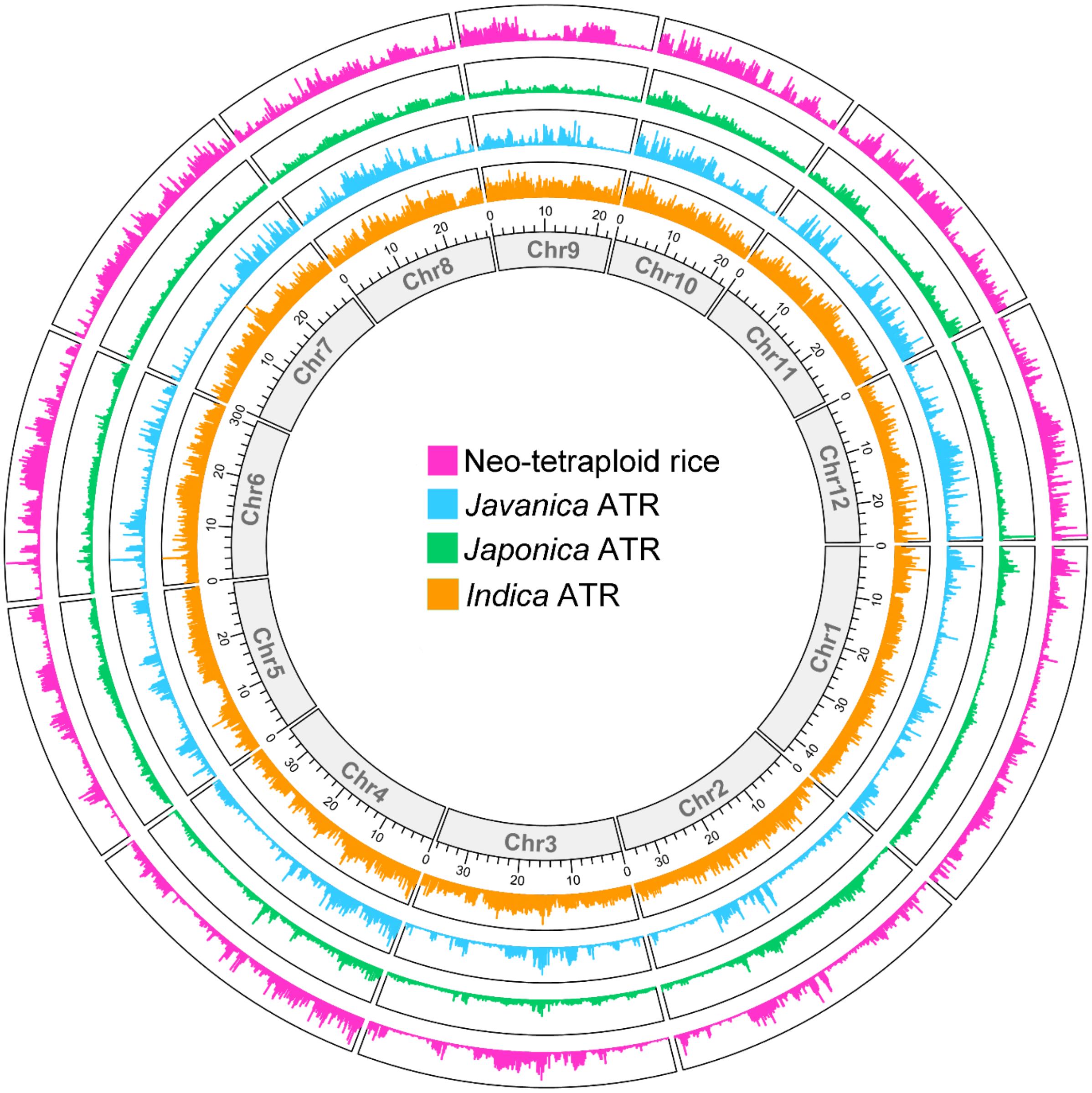

Figure S1. Circos plot illustrating the variation (including SNPs and InDels) density of different types of tetraploid rice against MSU7 reference genome.

ATR represent autotetraploid rice.

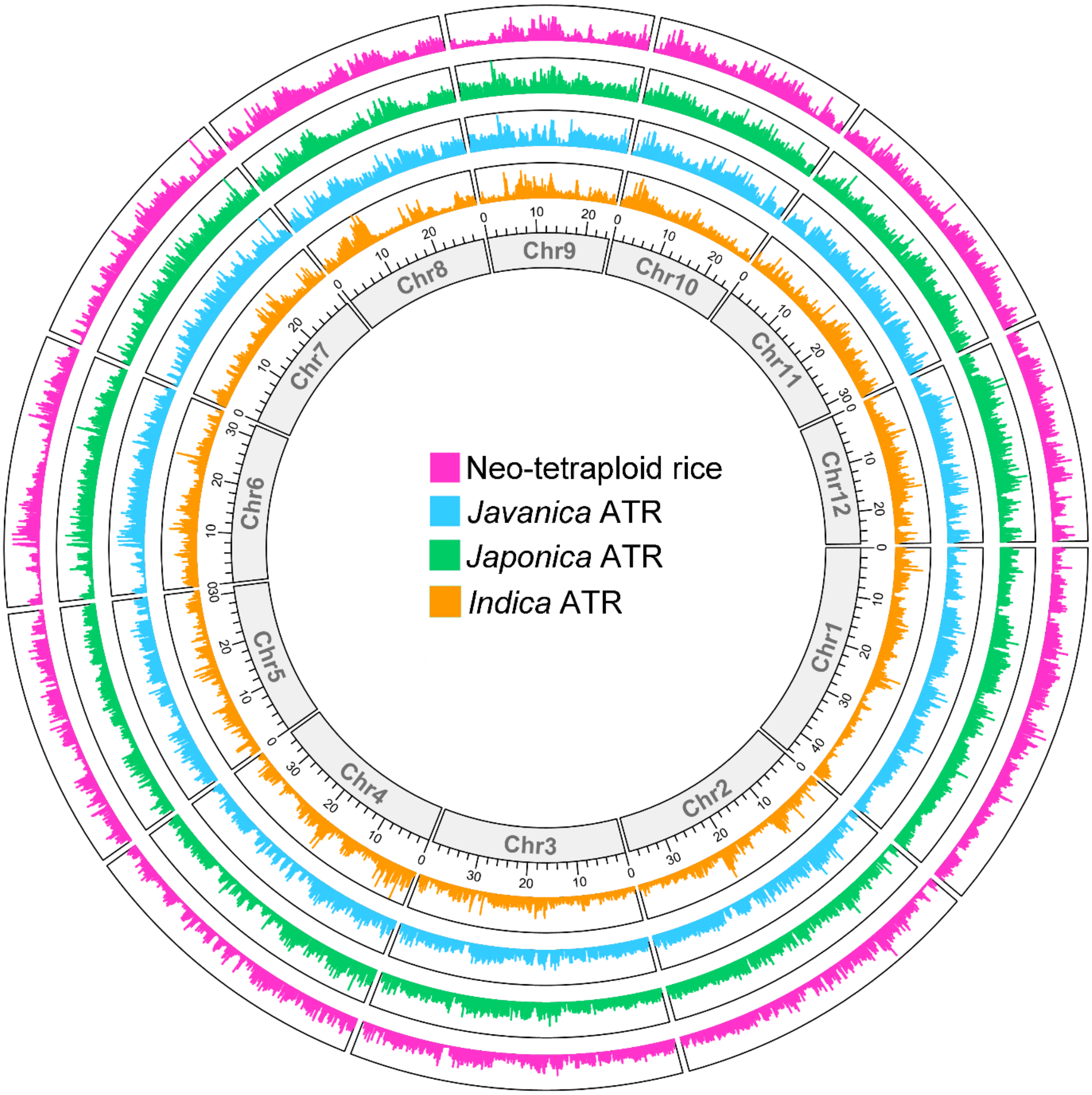

Figure S2. Circos plot illustrating the variation (including SNPs and InDels) density of different types of tetraploid rice against R498 reference genome.

ATR represent autotetraploid rice.

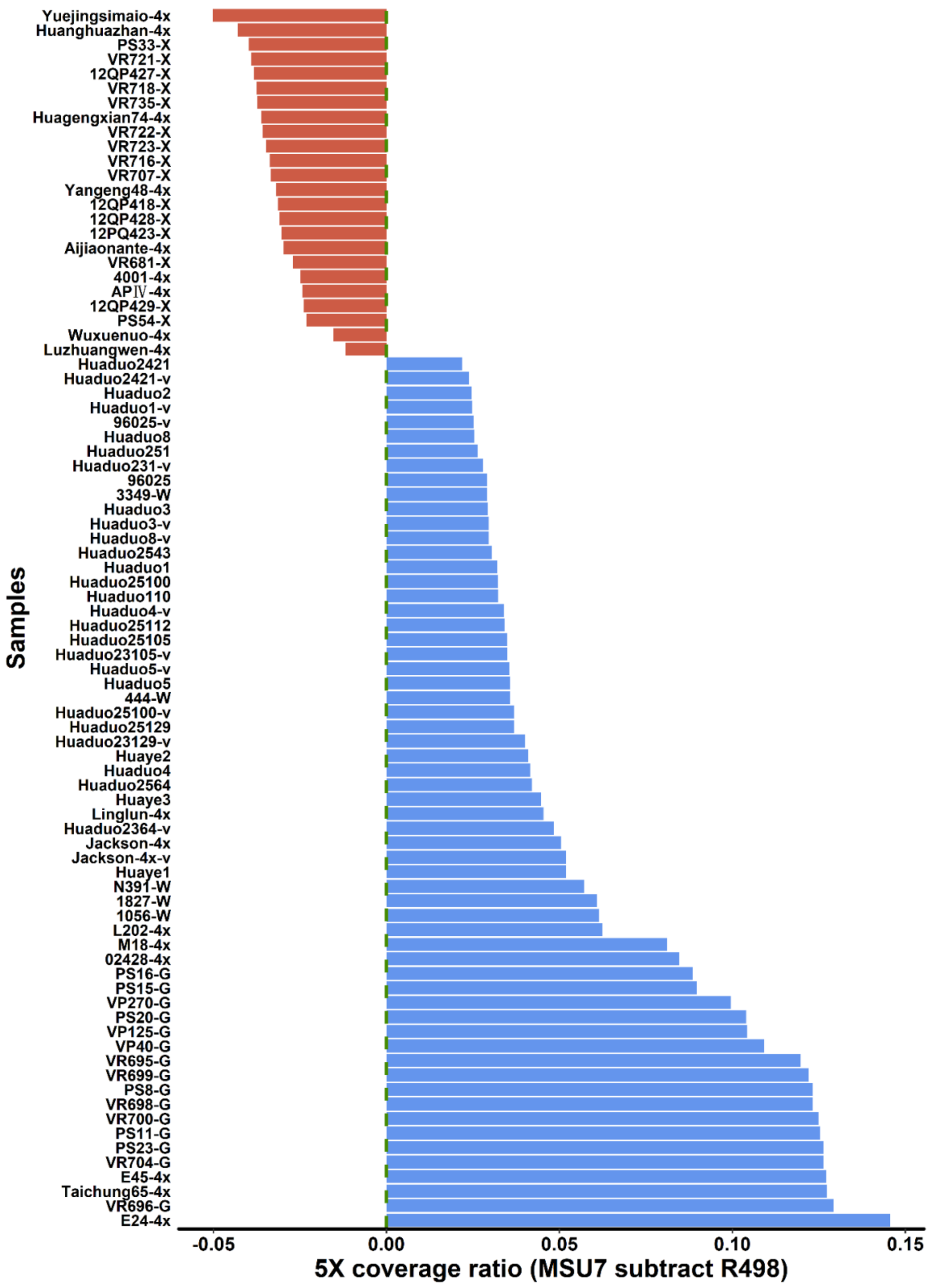

Figure S3. Difference of 5-fold mapping coverage ratio between *japonica* reference genome MSU7 and *indica* reference genome R498.

X-axis indicates subtraction values between the two genomes.

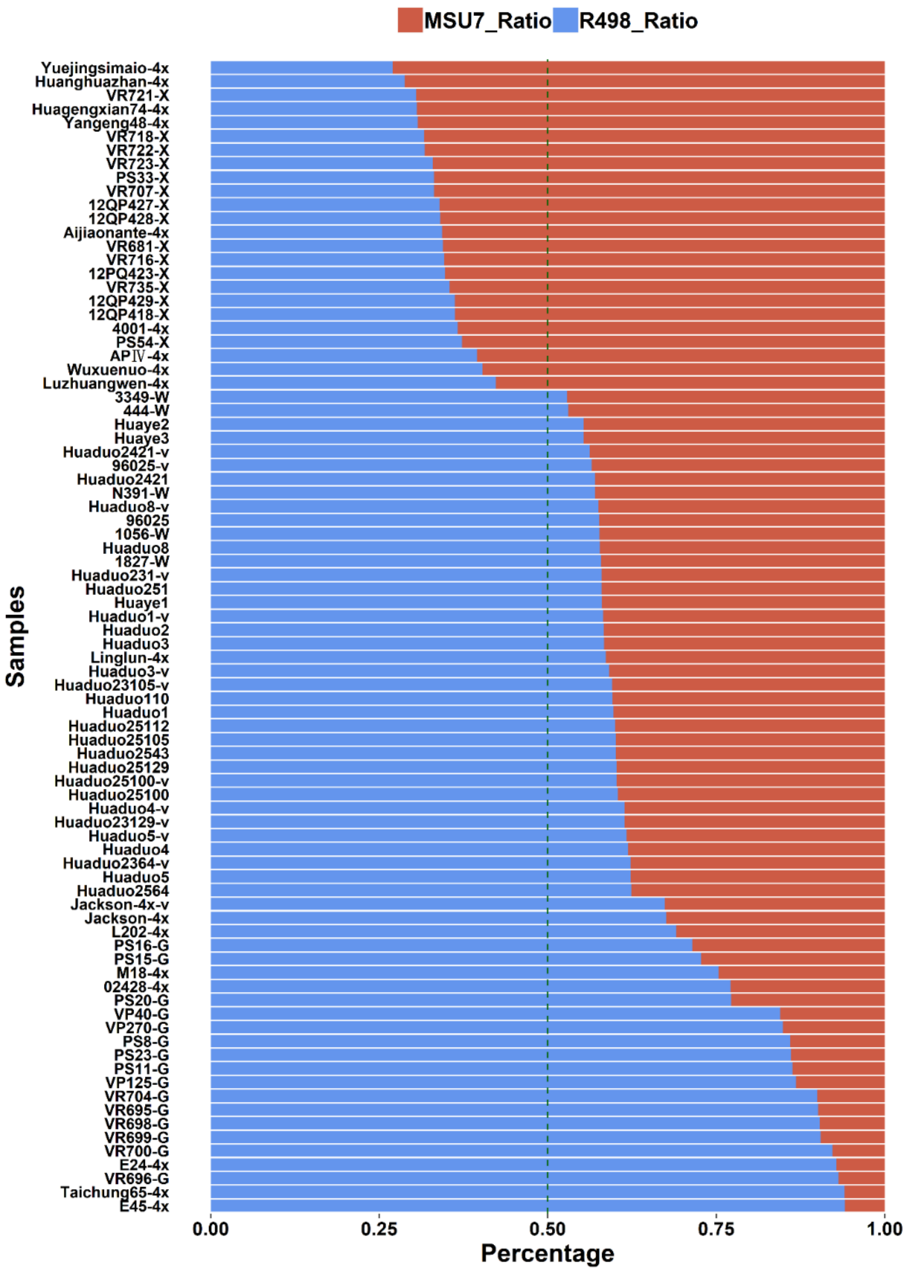

Figure S4. Comparison of genomic variation counts that generated based on *japonica* reference genome MSU7 and *indica* reference genome R498. The ratio between genomic variations were counted by two reference genomes shown on the x-axis.

X-axis indicates the ratio of genomic variation counts between two genomes.

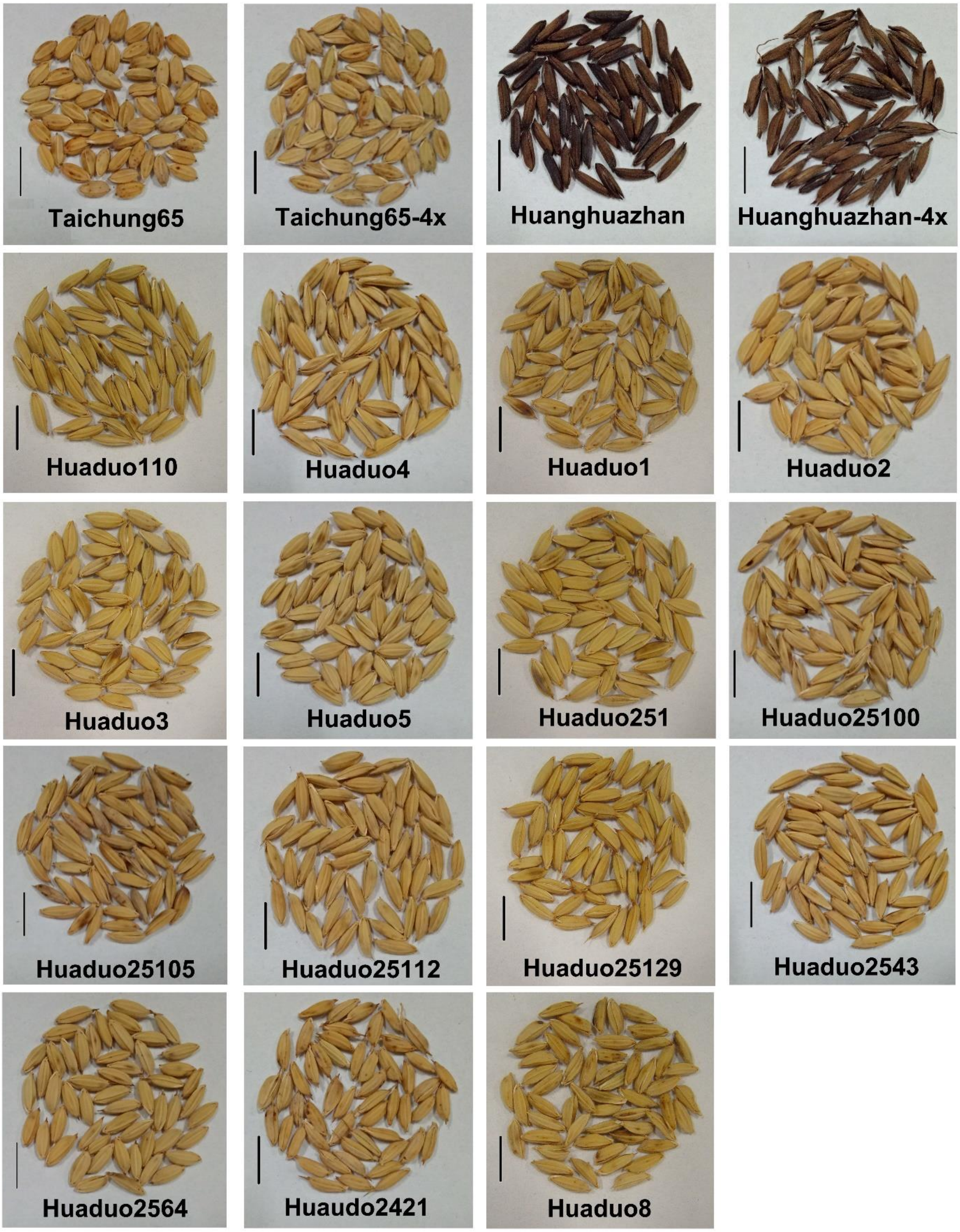

Figure S5. Phenol reaction of neo-tetraploid rice lines.

The *indica* autotetraploid Huanghuazhan-4x and its diploid counterpart and *japonica* autotetraploid Taichung65-4x and its diploid counterpart were used as controls. Bar size = 1 cm.

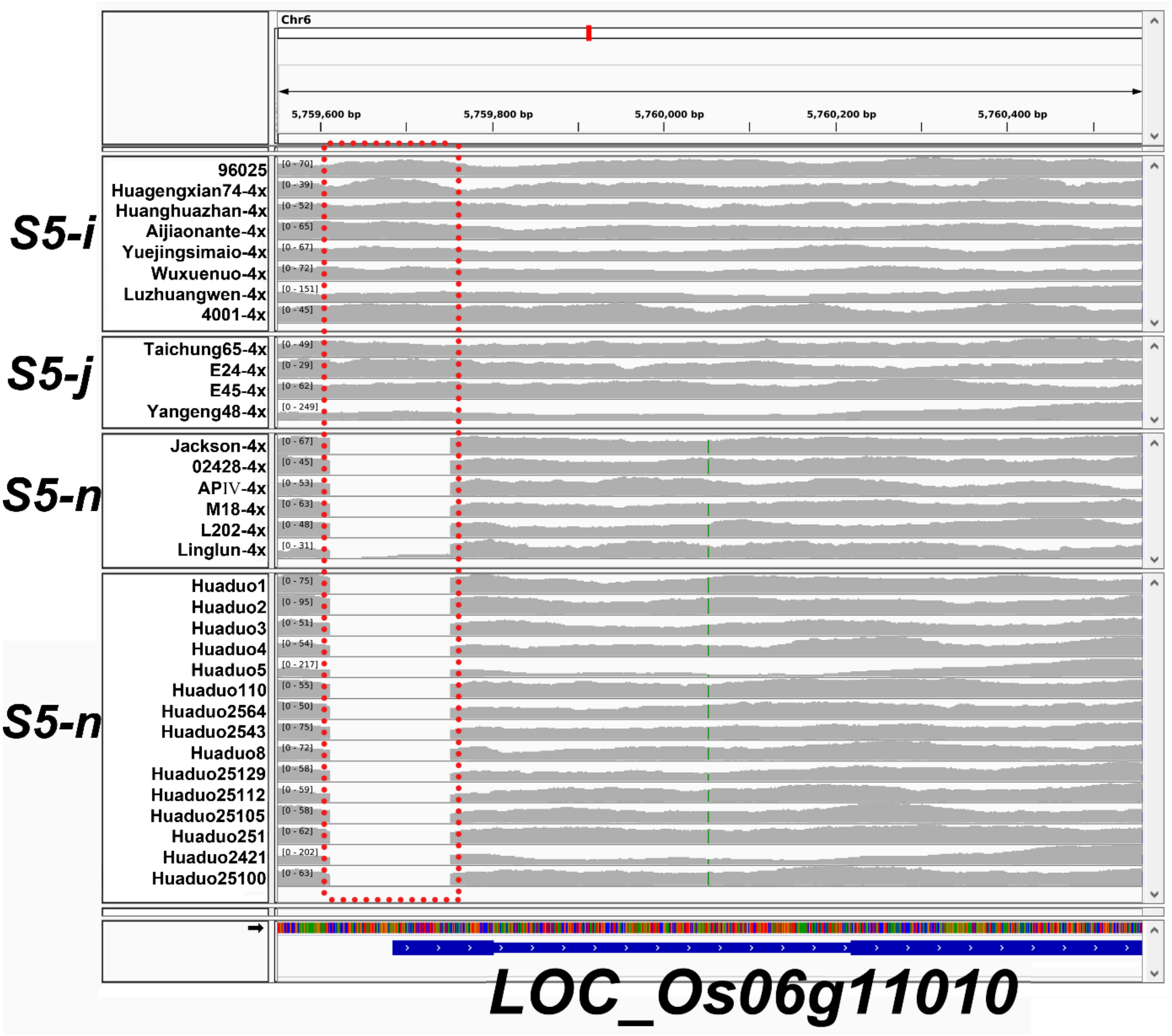

Figure S6. Sequencing coverage of wide compatibility gene *S5* (*LOC_Os06g11010*) in autotetraploid rice lines and neo-tetraploid rice lines.

Sequencing coverage was illustrated using IGV software. The region that not covered by sequencing reads showed the deletion of S_5^n gene.

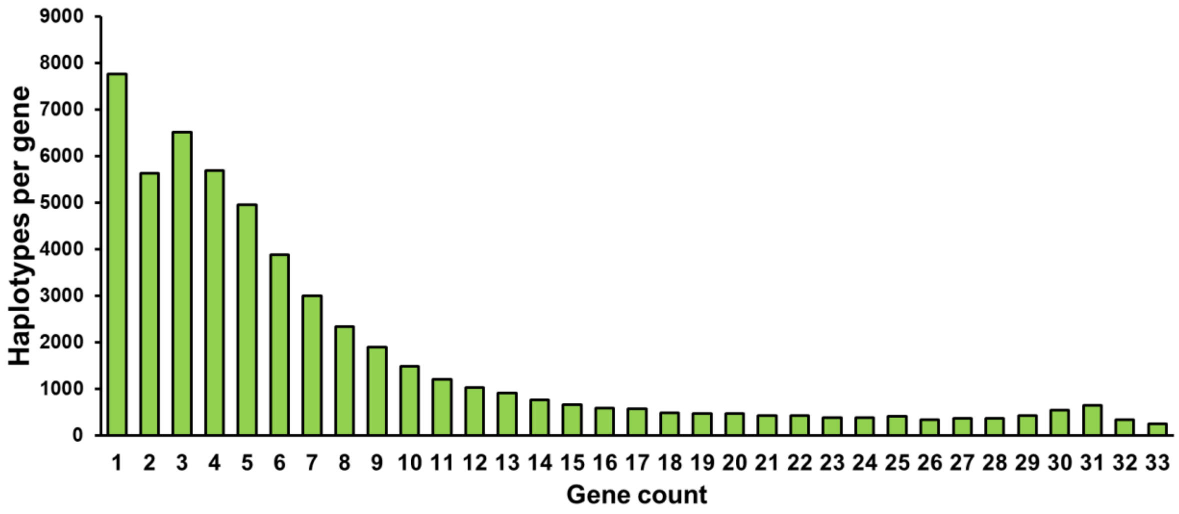

Figure S7 Distribution of haplotypes per gene of the 55801 annotated genes in tetraploid rice.

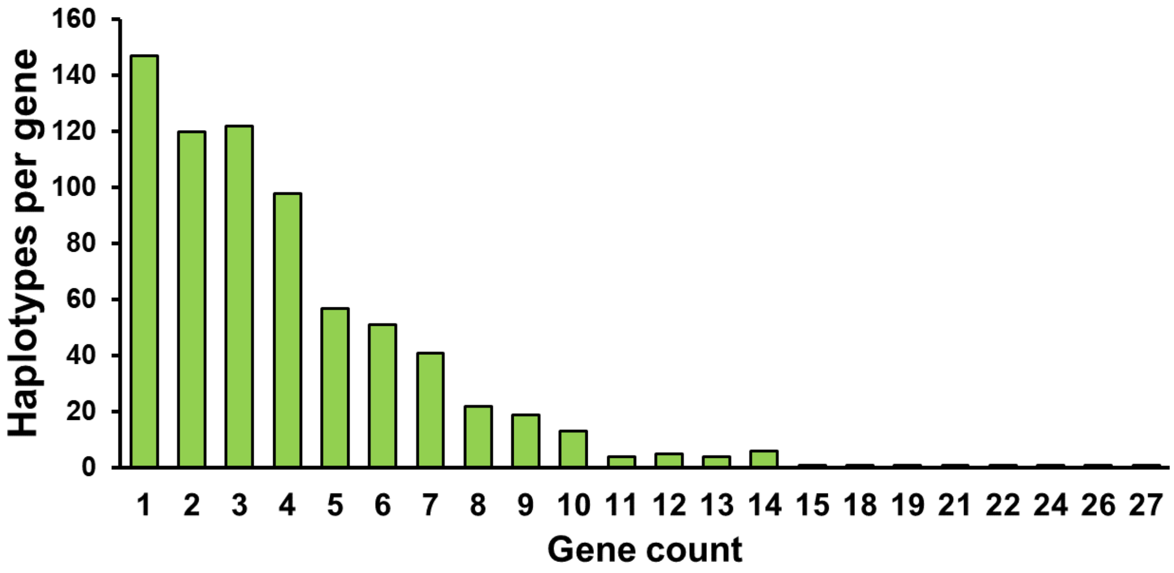

Figure S8. Distribution of haplotypes per gene in the 717 agronomically important genes in tetraploid rice.

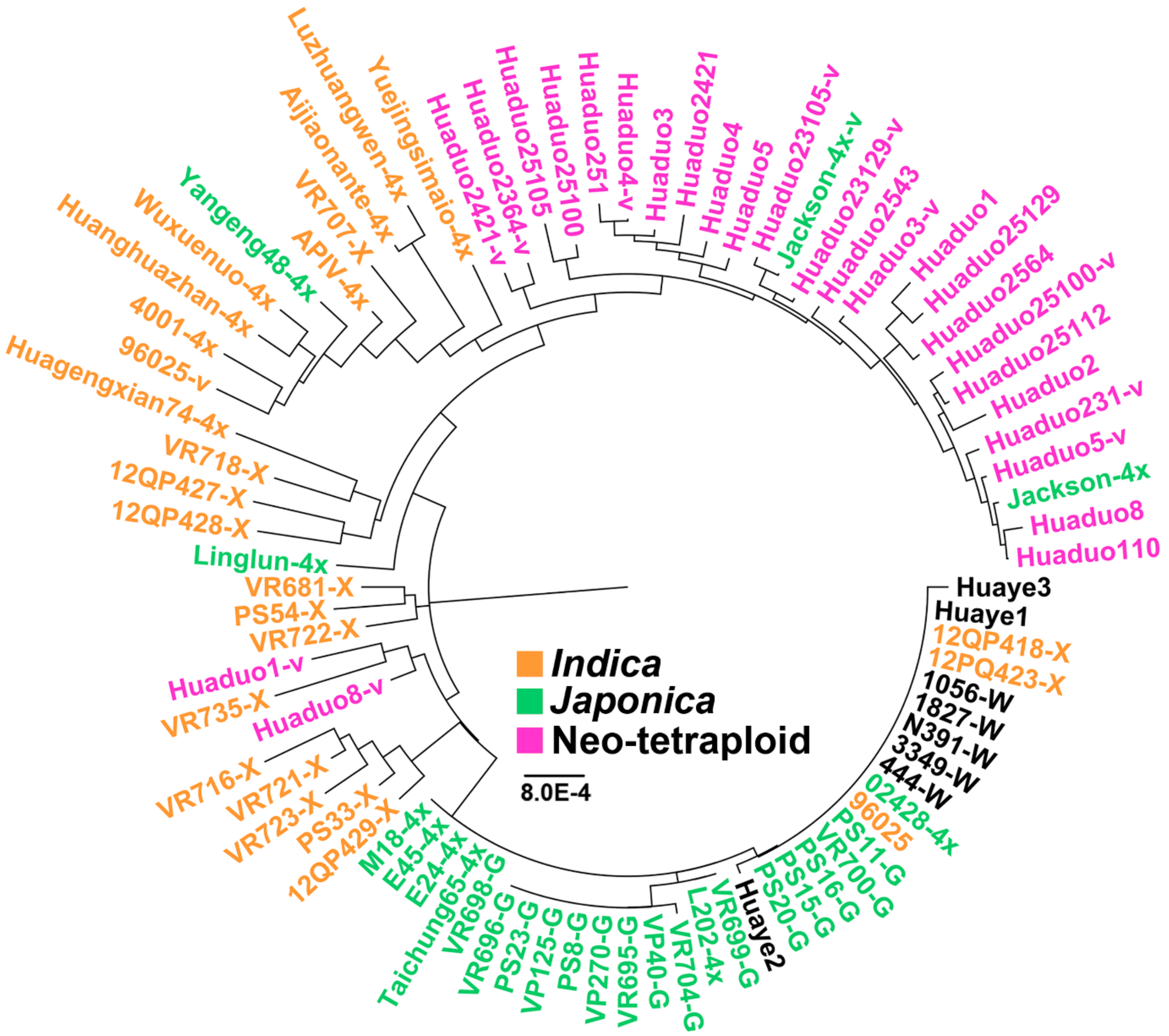

Figure S9a. The phylogenetic tree of *LOC_Os04g09310* in autotetraploid rice and neo-tetraploid rice lines.

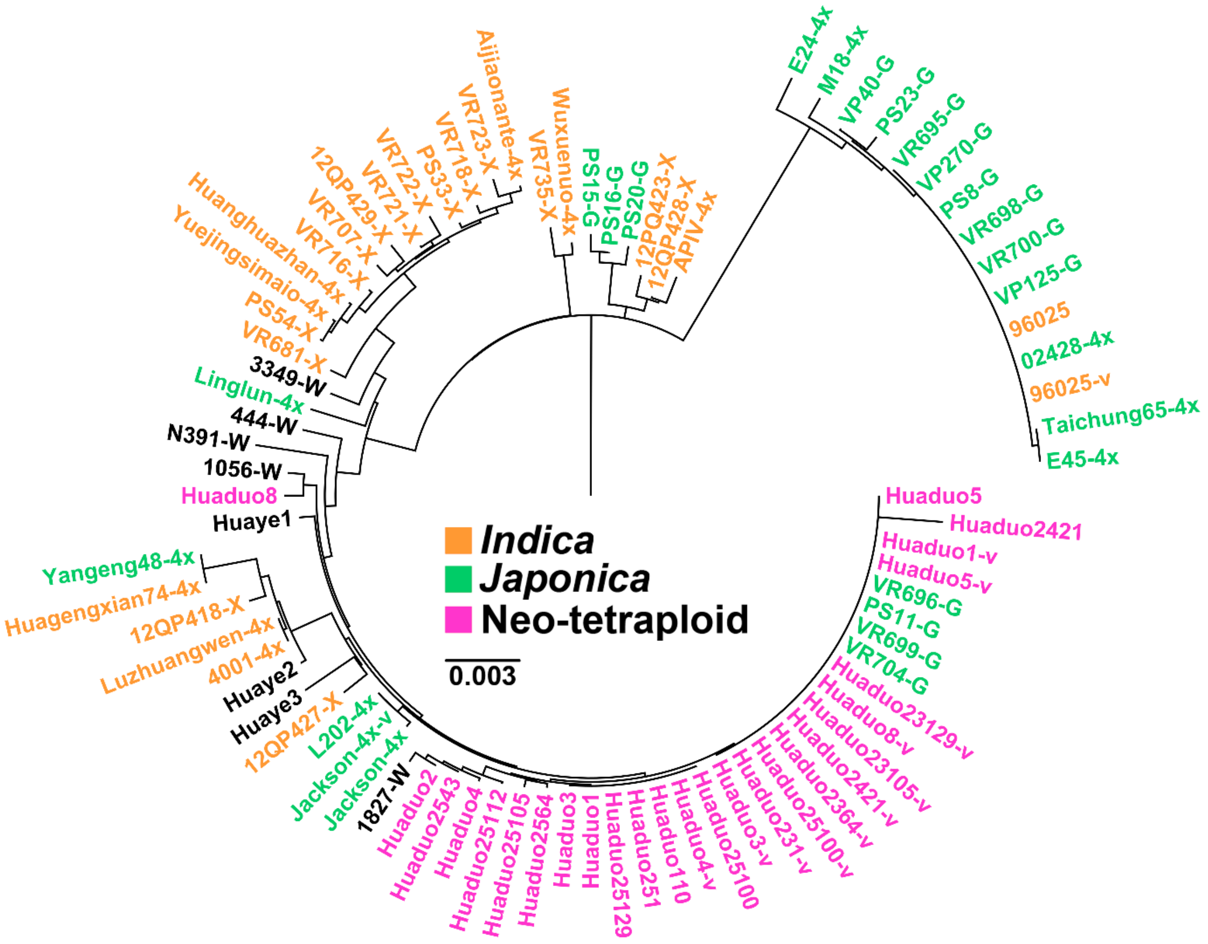

Figure S9b. The phylogenetic tree of *LOC_Os05g02840* in autotetraploid rice and neo-tetraploid rice lines.

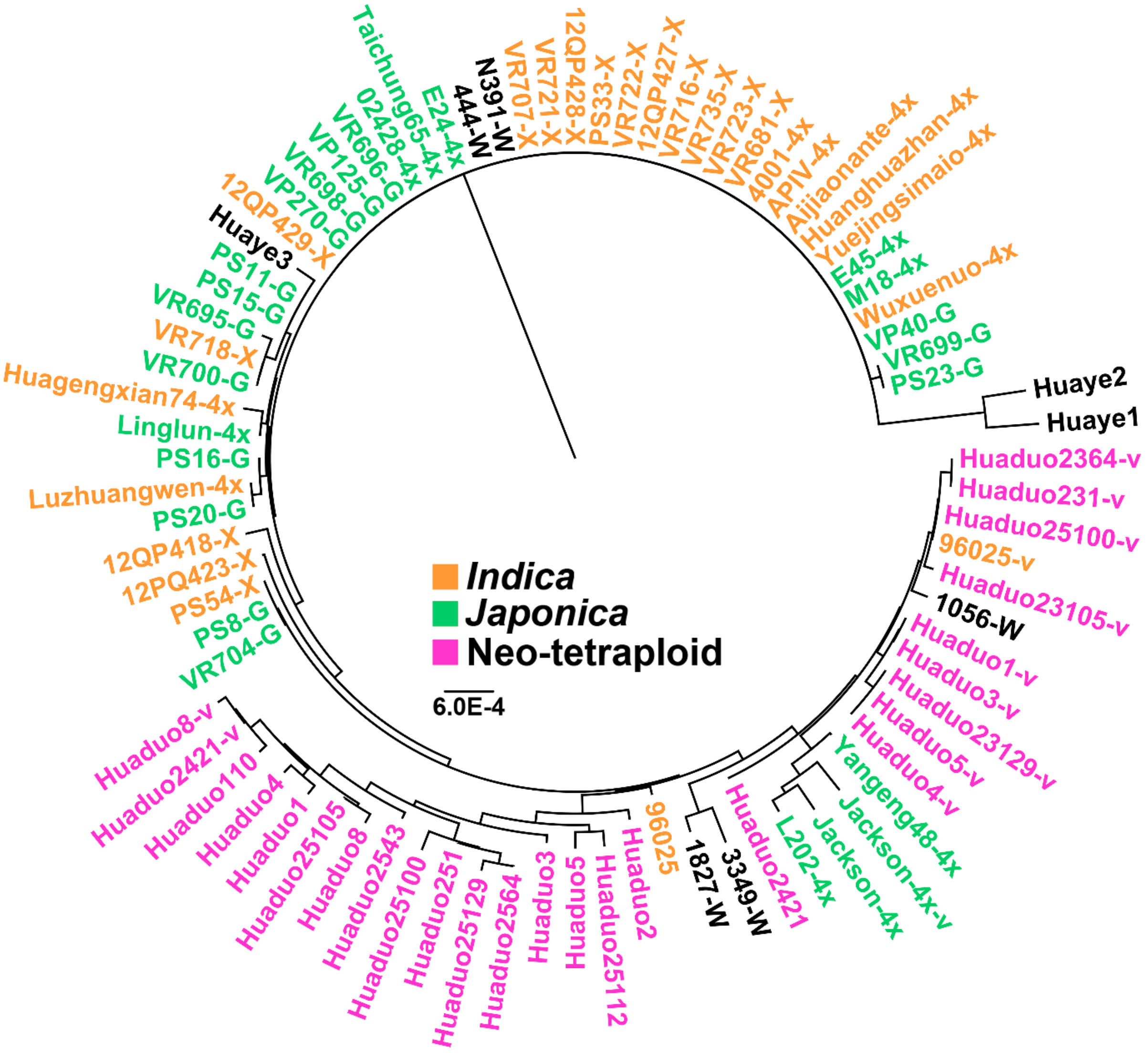

Figure S9c. The phylogenetic tree of *LOC_Os05g43000* in autotetraploid rice and neo-tetraploid rice lines.

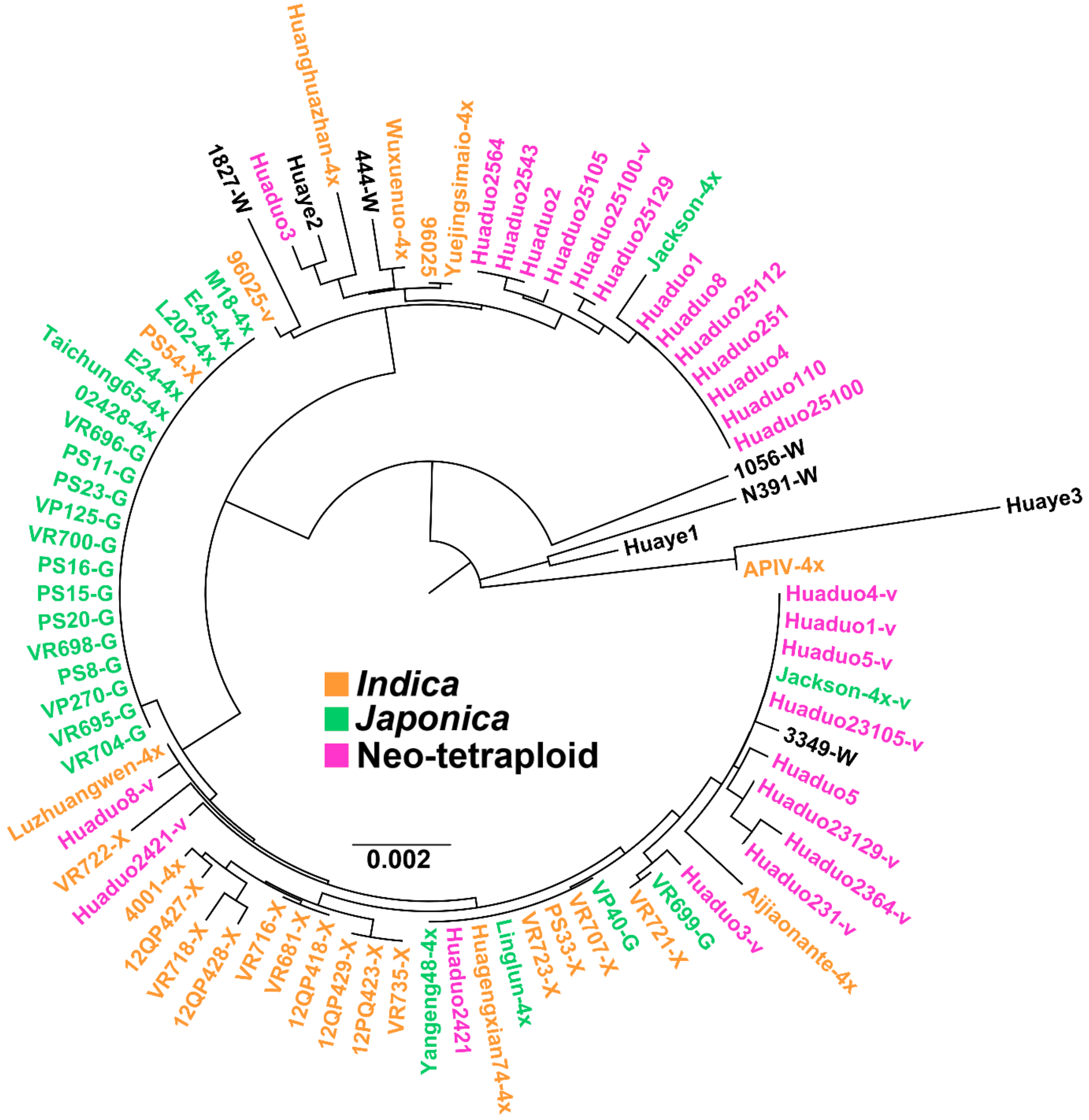

Figure S9d. The phylogenetic tree of *LOC_Os10g25730* in autotetraploid rice and neo-tetraploid rice lines.

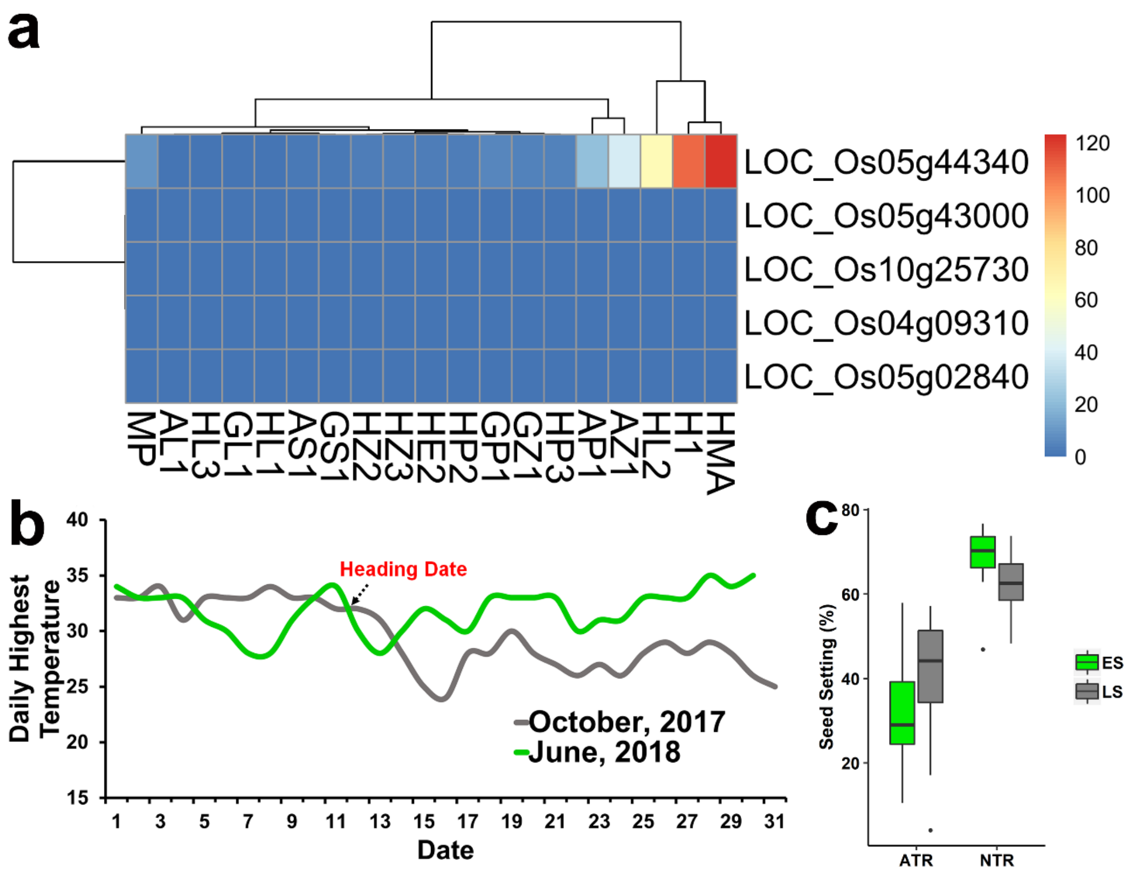

Figure S10. The expression pattern analysis of *HSP101* and the impact of temperature during flowering in tetraploid rice lines.

(a) The expression analysis of *HSP101* in different tissues of neo-tetraploid rice using RNA-Seq data. (b) Daily highest temperature distribution during heading stage in late season 2017 and early season 2018 in Guangzhou. (c) Comparison of seed setting between autotetraploid rice (ATR) lines and neo-tetraploid rice (NTR) lines under different temperatures during early and late seasons.

MP, young panicle shorter than 5 mm; AL1, flag leaf before flowering stage; HL3, flag leaf at 3 days after flowering (DAF); GL1, flag leaf at 5 DAF; HL1, flag leaf at meiosis stage; AS1, spikelet before flowering; GS1, spikelet at 5 DAF; HZ2, sheath at flowering stage; HZ3, sheath at 3 DAF; HE2, ovary at flowering stage; HP2, anther at flowering stage; GP1, sheath at 5 DAF; GZ1, branch and stem of panicle at 5 DAF; HP3, ovary at 3 DAF; AP1, sheath at flowering stage; AZ1, branch and stem of panicle at flowering stage; HL2, flag leaf at flowering stage; H1, anther at meiosis stage; HMA, anther and ovary at meiosis stage.

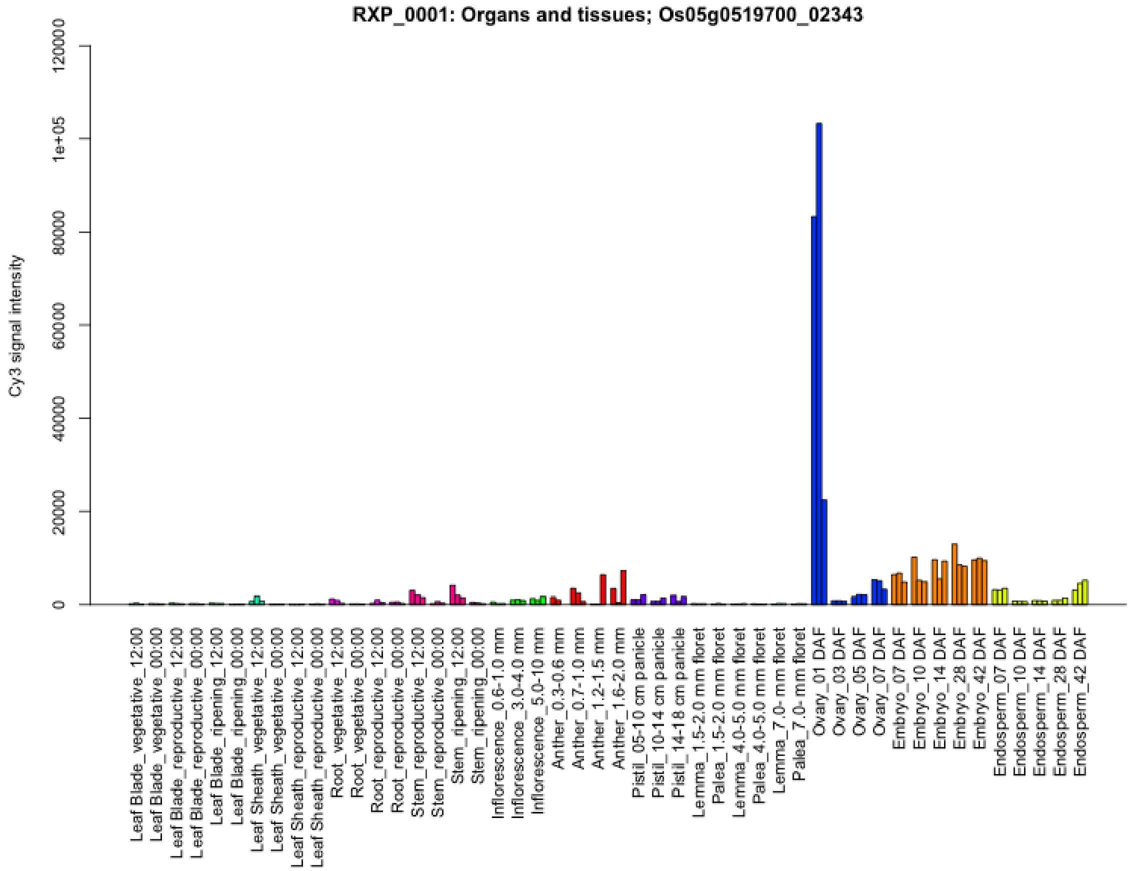

Figure S11. The expression pattern analysis of *HSP101* in diploid rice Nipponbare using the RiceXPro database.

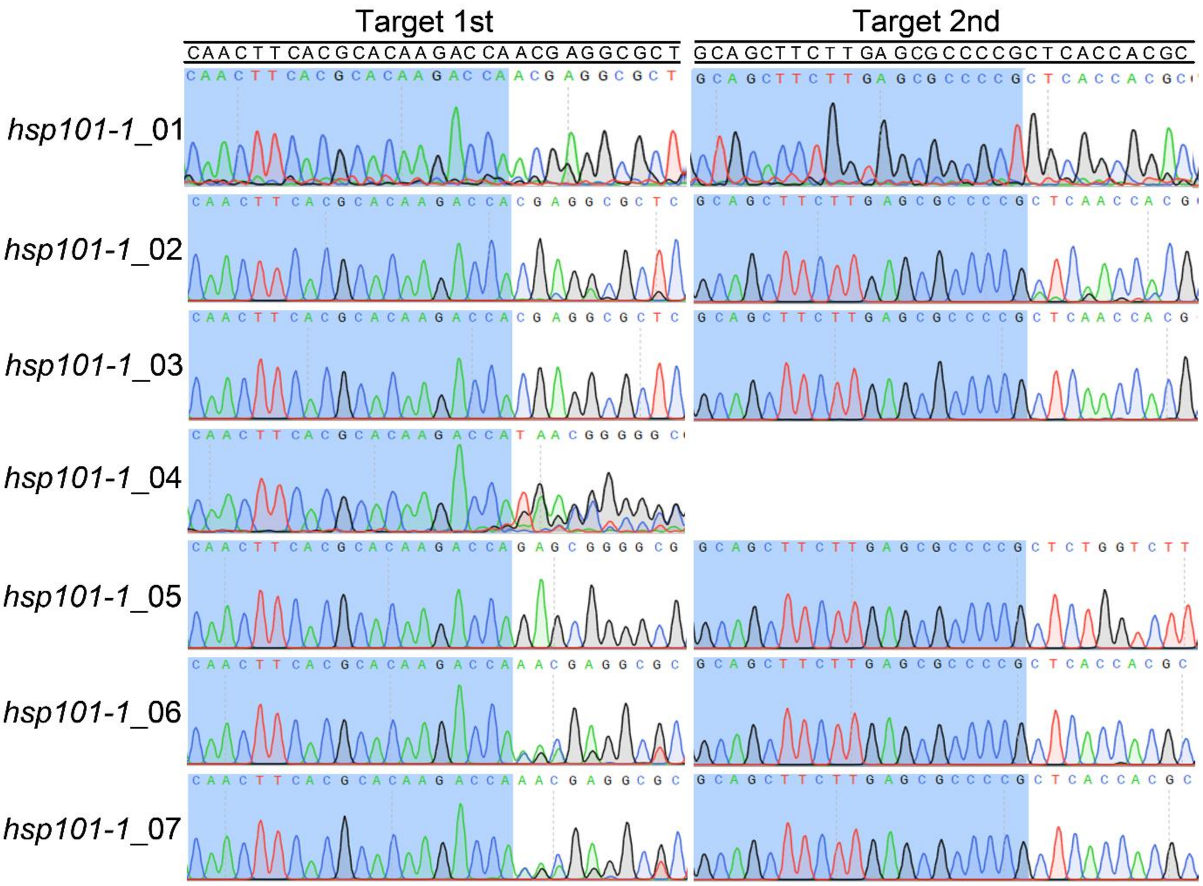

Figure S12. Sanger sequencing chromatogram of *hsp101-1* mutants.

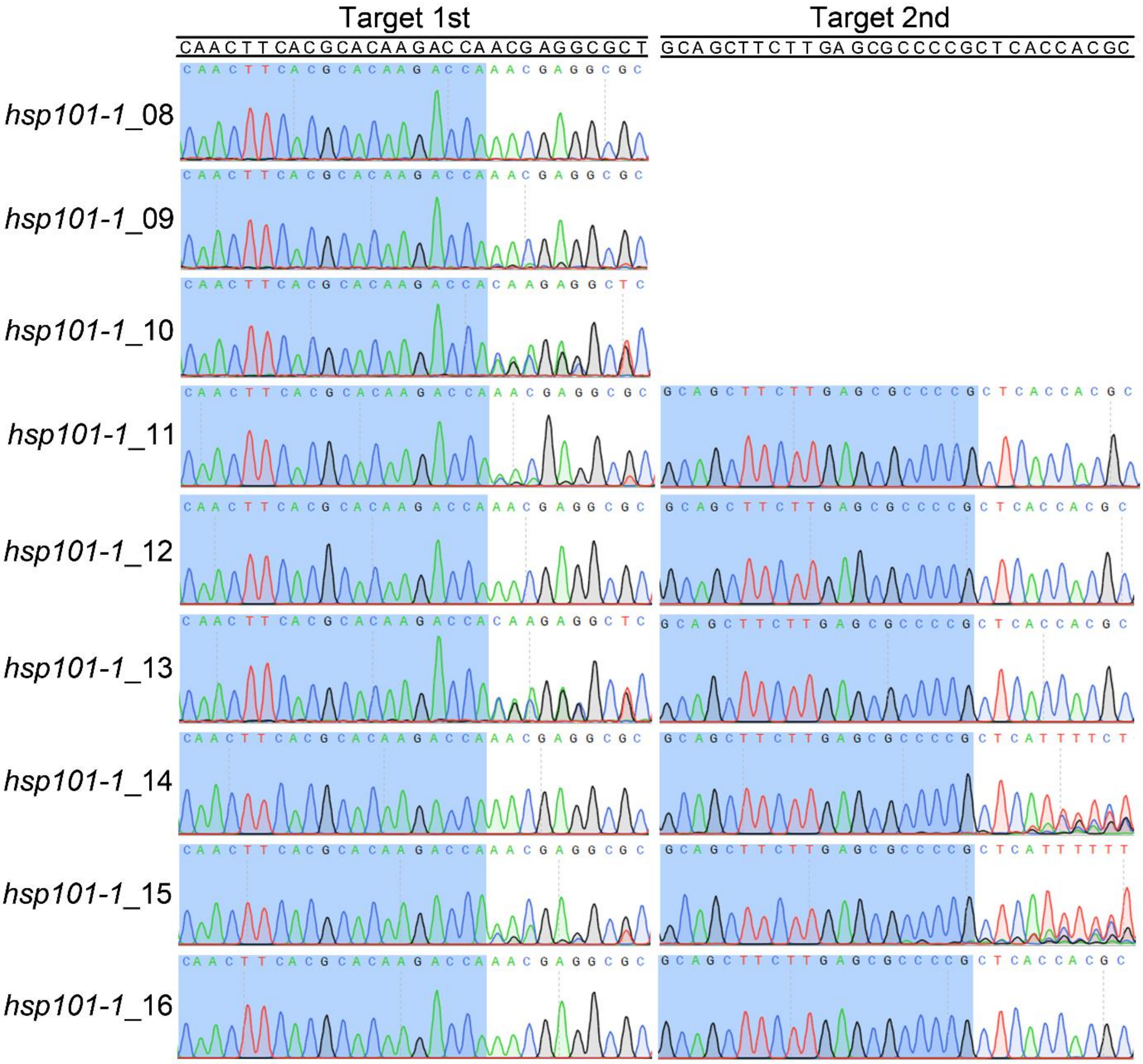

Figure S12. Sanger sequencing chromatogram of *hsp101-1* mutants (continued).

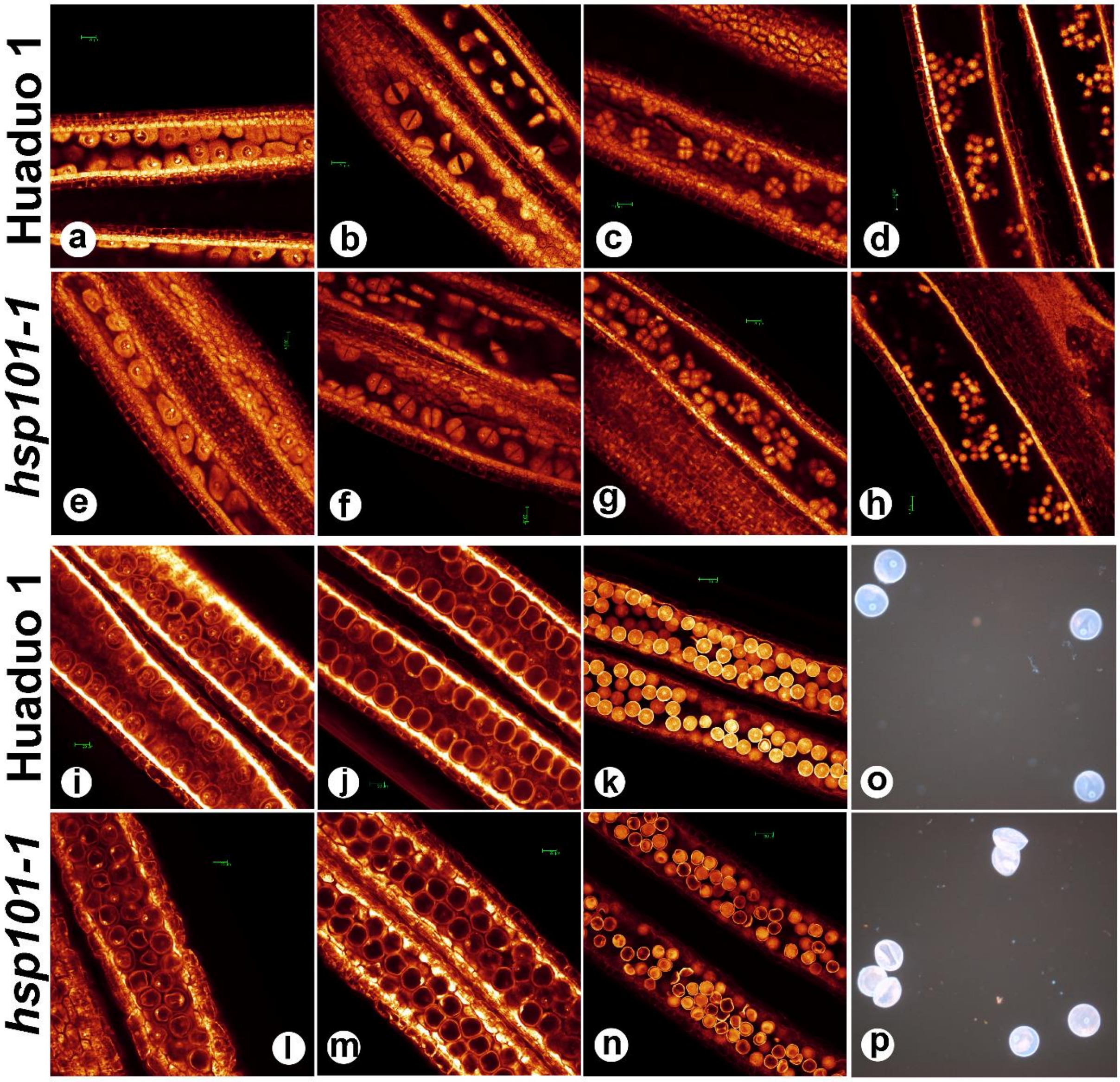

Figure S13 Cytological observation of pollen development of *hsp101-1* and wild type Huaduo 1. (a, e) prophase I; (b, f) dyad stage; (c, g) tetrad stage; (d, h) early microspore stage; (i, l) late microspore stage; (j, m) bicellular pollen stage; (k, n) mature pollen stage; (o, p) late microspore stage stained by DAPI (4,6-diamidino-2-phenylindole).
